# Supporting patient self-management: A cross-sectional and prospective cohort study investigating Patient Activation Measure (PAM) and Clinician Support for PAM scores as part of a multi-centre haemodialysis breakthrough series collaborative

**DOI:** 10.1371/journal.pone.0303299

**Published:** 2024-05-22

**Authors:** Maria Keriakos, Sonia Lee, Catherine Stannard, Steven Ariss, Louese Dunn, Martin Wilkie, James Fotheringham

**Affiliations:** 1 School of Health and Related Research, ScHARR, University of Sheffield, Sheffield, England; 2 Sheffield Kidney Institute, Sheffield Teaching Hospitals NHS Foundation Trust, Sheffield, England; 3 UK Renal Registry, Bristol, England; Monash University, AUSTRALIA

## Abstract

**Background:**

Patient self-management, measured by the Patient Activation Measure (PAM), is associated with reduced healthcare utilisation and better health-related quality of life. Self-management in haemodialysis (HD) is challenging and may require support from clinicians with positive attitudes towards self-management, measured by the Clinician Support for PAM (CSPAM).

**Objectives:**

To assess whether kidney staff CSPAM scores are: 1) associated with their centre’s patient PAM scores and 2) modifiable through staff coaching.

**Methods:**

Baseline PAM and CSPAM and six-month CSPAM were collected from HD patients and kidney staff respectively in seven UK kidney centres as part of a six-month breakthrough series collaborative (BTSC), which trained kidney staff in supporting patient independence with HD tasks. Firstly, multivariable linear regression analyses adjusted for patient characteristics were used to test the baseline association between centre-level staff CSPAM scores and patient PAM scores. Secondly, paired univariate and unpaired multivariable linear regression analyses were conducted to compare staff CSPAM scores at baseline and six months.

**Results:**

236 PAM questionnaires (mean score = 55.5) and 89 CSPAM questionnaires (median score = 72.6) were analysed at baseline. There was no significant association between centre-level mean CSPAM scores and PAM scores in univariate analyses (P = 0.321). After adjusting for patient-level characteristics, increasing centre-level mean CSPAM score by 1 point resulted in a non-significant 0.3-point increase in PAM score (0.328 (95% CI: -0.157 to 0.812; P = 0.184). Paired (n = 37) and unpaired (n = 174) staff analyses showed a non-significant change in CSPAM scores following the BTSC intervention (mean change in CSPAM score in unpaired analysis = 1.339 (95% CI: -1.945 to 4.623; P = 0.422).

**Conclusions:**

Lack of a significant: 1) association between CSPAM and PAM scores and 2) change in CSPAM scores suggest that modifying staff beliefs alone is less likely to influence patient self-management, requiring co-production between patients and staff.

## Introduction

Over 80% of patients with kidney failure who require long-term dialysis, receive in-centre haemodialysis (HD) which is generally delivered three times per week for about four hours [1]. The current organisation of in-centre HD means patients often assume a passive role due to staff time pressures [2], insufficient patient education [3] and the traditional, paternalistic healthcare culture [2–5].

It is well recognised that supporting patient self-management is integral to caring for patients with long-term conditions and is prioritised in healthcare policy [6, 7]. Patient activation, measured by the Patient Activation Measure (PAM) is defined as the knowledge, skills and confidence a patient has towards self-management and is associated with improved health-promoting behaviours [8–10], satisfaction with care [11], and significantly lower healthcare utilisation rates [12]. Within kidney care, higher activation is associated with reduced depression and anxiety and higher quality of life [13]. However, one-third of patients with kidney failure in England are in the lowest level of activation [14], compared to only 8% of adults with chronic conditions nationally [15]. Patient activation decreases with increasing chronic kidney disease (CKD) severity in the non-dialysis population [16]. Amongst patients with end-stage kidney disease, in-centre HD patients have the lowest patient activation [13]. Home haemodialysis (HHD), a prime example of self-management, is associated with higher patient activation scores [17], quality of life and survival rates and reduced hospitalisation [18] and is cost-effective, saving around £12,000 per patient per year in comparison to in-centre HD [19].

It is not yet known whether staff attitudes can be harnessed to improve patient activation specifically in the unique context of HD, where there is high staff contact time and potentially large gains in cost-effectiveness, survival and quality of life. We are also not aware of any studies to date that have investigated whether staff attitudes towards patient self-management can be modified, using the Clinician Support for Patient Activation Measure (CSPAM) before and after an intervention. A multicentre study in England conducted across patients with kidney failure as a whole found no association between CSPAM and PAM, but did not focus on the unique area of in-centre HD care [13]. However, cross-sectional studies involving patients with other chronic illnesses have found that patients who feel that their clinicians are person-centred or support them to self-manage are more confident and able to self-manage than those who do not [4, 20]. These studies suggest that there could be benefit in modifying staff attitudes to support patient self-management.

Shared HD care is a person-centred way of delivering in-centre HD where patients have the choice and opportunity to participate in their HD tasks (S1 Table) [21], with the broader aim of changing HD culture to actively involve patients and work in partnership. Shared HD care transforms staff into self-management educators and requires a shift in attitudes and behaviours, aligning with the Transtheoretical model, to prioritise routine patient engagement instead of seeing this as an additional task [2, 22, 23]. Staff report greater satisfaction and stronger patient rapport, with more time for those in need [2, 24, 25]. Patients are valued as equal partners and feel empowered to voice their concerns, preferences and experience in shared decision-making and in co-producing the design and delivery of shared HD care [22]. Patients learn how to problem solve, understand and inquire more about their treatment and biochemical results and experience control, independence and a sense of achievement [2, 26].

The SHAREHD intervention is a quality improvement (QI) programme, utilising the Breakthrough Series Collaborative (BTSC) QI methodology to support kidney centres to implement shared HD care. A previous stepped wedge cluster randomised controlled trial, utilising the SHAREHD intervention, was associated with significant increases in number of HD tasks performed and HHD uptake in those performing less than five HD tasks at baseline and significant reductions in hospitalisations due to fluid overload, suggesting improved home self-management [27]. The intervention had the largest impact on patients in the lowest PAM levels, who significantly improved their PAM scores, though overall PAM scores were unchanged. The SHAREHD intervention’s impact on staff attitudes was not assessed.

In this stand-alone six-month SHAREHD BTSC, we first collected baseline cross-sectional in-centre HD patient PAM and HD staff CSPAM data from seven participating UK centres, hypothesising that staff attitudes towards self-management may be associated with their HD patients’ ability to self-manage at their centre. This is because higher CSPAM scores are associated with positive staff behaviours that may influence patient self-management ability [28, 29]. Secondly, we collected staff CSPAM data from the same participating centres post-intervention (at six months), hypothesising that longitudinal HD staff attitudes towards self-management may change by participating in the empowering and supportive shared HD care movement and seeing first-hand the beneficial patient impact that can be made within their own resource-tight settings, potentially eroding long-held beliefs on care delivery [22]. If HD staff attitudes influence HD patient self-management and such staff attitudes can be modified, the case for implementing staff coaching to support patient self-management is strengthened.

## Methodology

### Study design

This study was conducted across seven UK kidney centres and collected cross-sectional, baseline in-centre HD patient data, including PAM, and longitudinal HD staff data, including CSPAM, at baseline and after a stand-alone six-month BTSC, known as SHAREHD. The UK Renal Registry (UKRR) facilitated this study and were using NHS England’s commercial licences for the PAM and CSPAM. Two separate processes were utilised to ensure ethical use of data, one for patient data collection and another for staff data collection. Patients were informed that by completing the questionnaire, they consented to their data being held by the UKRR and their renal unit. Although primary patient data was collected as part of quality improvement activity, the UKRR also holds permissions under section 251 of the NHS Act 2006, to gather, process, and share confidential patient information for the purposes of audit and research. These permissions are renewed annually by the UK’s Health Research Authority’s Confidentiality Advisory Group. Ethical approval for staff data collection was obtained on 4^th^ May 2018 from the University of Sheffield Research Ethics Committee as administered by the medical school (approval number: 019561). Individual staff participation was informed consent after reading the research information on the front of each CSPAM questionnaire which stated the purpose for which the data was being collected.

### Study setting and participants

UK kidney centres voluntarily applied to participate in SHAREHD between January and February 2018. Seven kidney centres were selected on the basis of enthusiasm and commitment to shared HD care. The following centres and their associated satellite units (numbered in brackets) participated: Belfast City Hospital (no satellites), Queen Elizabeth Hospital in Birmingham (10 satellites), University Hospital Crosshouse in Kilmarnock (no satellites), James Cook University Hospital in Middlesborough (three satellites), Royal Liverpool University Hospital (four satellites), Salford Royal Hospital in Greater Manchester (four satellites) and Western Trust in Altnagelvin (no satellites). Belfast, Birmingham and Liverpool are the only participating kidney transplant centres. HD care in the UK is provided by both the NHS and private companies and therefore participating satellite units are either NHS-run or privately-run in partnership with their main NHS centre. Privately-run satellite units train their own nursing staff and have different policies, targets and managerial structure that mean the approach to implementing shared HD care may differ from that of NHS units. However, all main centres and their satellite units have regular meetings where shared HD care could be discussed.

This study ran from April 2018 –March 2019, with the first participants being recruited in April 2018, and was nested within the SHAREHD quality improvement collaborative. One staff member at each kidney centre was assigned the role of staff champion for local patient and staff data collection. Staff champions agreed to circulate at least 50 paper copies of patient questionnaires, with more copies provided on request. Adult incident and prevalent in-centre HD patients were recruited into the study between April and May 2018 by completing the patient questionnaire. Patients could complete the questionnaire on their own or with assistance to increase participation in groups that may not otherwise have been represented, such as those with reduced health literacy or any individual need. Staff champions also agreed to circulate at least 10 paper CSPAM questionnaires to kidney staff with a variety of roles in their centre. By implementing shared HD care, we hypothesised that the workplace culture would change and therefore staff were sampled from the participating centres and not just from staff who attended the learning events. Kidney staff were recruited into the study at baseline (June—September 2018) and post-intervention six months later (December 2018—March 2019) by completion of the staff CSPAM questionnaire. Sampling the same staff at both time periods was advised to enable comparison.

### Data collection and procedures

Information sheets explaining patient and staff data collection were distributed to staff champions. Completed patient questionnaires were securely transported to the UKRR for data inputting and sent to Insignia Health for PAM scoring. Anonymised patient data was fed back to kidney centres. Patients had the opportunity to review their data via the online portal ‘PatientView’ [30]. Identifiable patient data captured in the patient questionnaires was shared with the SHAREHD QI programme to facilitate patient care and communicate with centres should concerning responses be identified. Researchers had access to NHS number and postcode.

On each page of the CSPAM questionnaires, a participant identification number consisting of renal centre number, respondent initials and collection round was used to identify the same individuals at first and second data collection. Staff champions were provided with a list of initials and job titles of baseline participating staff in their centre for repeat participation at six months. Completed staff questionnaires were sealed in an envelope by the respondent before being handed back to the staff champion and transported to the University of Sheffield for data inputting into an electronic database. The resultant electronic CSPAM responses were transferred to the UKRR and Insignia Health for scoring. Researchers had access to participating staff initials, gender, age group, job title and renal unit for data analysis and to assist with pairing responses. Though staff participants were not directly identifiable to researchers, small staff numbers within individual centres could theoretically make individuals identifiable. In addition, staff were given the informed choice of providing their email address for the sole purpose of receiving their individual scores if they wished. Staff champions could also request their centre-level summaries.

### Intervention

The SHAREHD quality improvement intervention was delivered using the evidence-based BTSC methodology developed by the Institute for Healthcare Improvement [21]. BTSCs achieve rapid improvements in quality of care by bringing together at least three members as ‘implementation leaders’ from each healthcare team to attend three face-to-face learning events, where they are coached on QI methodologies and share experiences of what worked and how to overcome challenges. Teams are supported to implement changes between learning events through conference calls with programme leads and online discussion forums to collaborate with peers. BTSCs have helped organisations reduce healthcare costs, hospitalisations and waiting times [21].

This stand-alone six-month SHAREHD BTSC was based on learning from a previous SHAREHD BTSC [27], which recognised that involving patients as partners, broadening teaching of QI methodology, sharing of site experiences and ownership of work improved the implementation of shared HD care. The intervention in this study was the implementation of shared HD care at the kidney centres using the BTSC methodology. The SHAREHD BTSC in this study consisted of three face-to-face learning events in June, October and December of 2018 (S2 Table). Learning events were attended by approximately five individuals as implementation leaders from each centre, including nurses, doctors and at least one patient representative. One attendee from each centre was designated the staff champion responsible for patient and staff questionnaire data collection. The implementation leaders were responsible for establishing and sustaining shared HD care at their unit. At each learning event, the aims of SHAREHD were summarised and kidney centres participating in the previous SHAREHD BTSC shared their experiences and advice. Implementation leaders were taught the ‘Model for Improvement’ QI curriculum [21], which helps teams design and share their own Plan-Do-Study-Act (PDSA) cycles at each learning event for iterative implementation at their centre. Methods of ‘spread’ (raising awareness) and ‘sustainability’ (maintaining changes) of shared HD care were discussed at learning events, while recognising that implementation follows a roadmap starting small with a few enthusiastic staff and following the stages of: 1) set-up, 2) testing, 3) maintenance and growth, 4) sustainability and rejuvenation and 5) standard practice. The first, second and third learning events covered the following themes respectively in relation to shared HD care: co-production with patients, communication with stakeholders and long-term sustainability of change (S2 Table). Practical examples of how each theme could be addressed in their local implementation plans were brainstormed and discussed to take back to their centres. Each site was encouraged to tailor their implementation approach to their local context supported by the consistent QI framework provided at learning events.

Staff who attended the learning events disseminated their learning at their sites through engaging stakeholders such as matrons, medical, managerial staff, patient representatives, patients and their families and carers. Communication tools and resources were provided to sites on the SHAREHD website to facilitate this [31]. Suggested approaches to raise awareness amongst staff and managers included discussion of benefits of shared HD care, patient stories and audit data at multidisciplinary team meetings including those with satellite units. Previous participating centres have also set up their own in-house shared HD care staff training programme. It was recommended that centres identify and address staff barriers to shared HD care at their centres early in the process. Shared HD care posters, banners, patient information leaflets and individualised HD task competency handbooks were used to raise awareness amongst patients, log their progress and encouraged them to ask questions during their dialysis such as: ‘Why is my weight important? How much fluid do I need to take off? What do my blood pressure numbers mean? Why are my tablets important?’ thereby additionally supporting home self-management. Waiting areas could be changed to incorporate weighing, blood pressure and hand washing stations and staff were encouraged to ask patients whether they would like to get involved in their dialysis and explain what they were doing while providing care. Previous participating centres have also utilised staff-patient coffee mornings, peer support and patient education videos to spread the message amongst patients and carers. Long-term sustainability was encouraged through inclusion of shared HD care in nursing handovers, patient notes, staff induction, roles and competencies, HHD programmes and as a modality choice in pre-dialysis clinics. The engagement of stakeholders, sustainability measures and celebration of achievements through publications and conference presentations aimed to promote staff cultural change so that shared HD care became standard practice.

**Fig 1:**
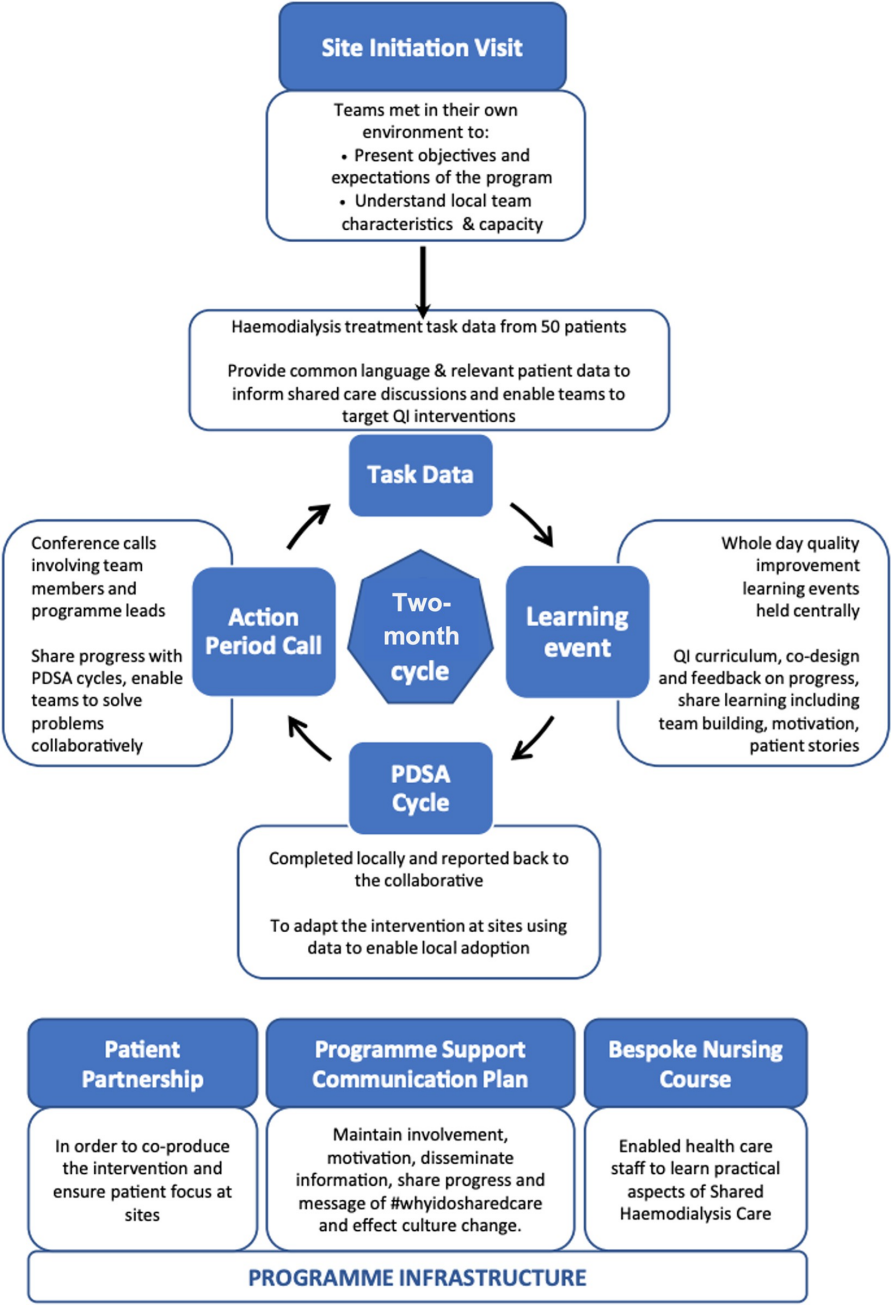
Diagram of the SHAREHD multi-centre Breakthrough Series Collaborative. Adapted from Fotheringham *et al*. [27].

Between learning events, sites were supported and progress tracked through site visits, phone calls, email correspondence, site logbooks and action period calls, which were conference calls attended by all teams and SHAREHD programme co-ordinators where progress with PDSA cycles were shared and problems were solved collaboratively (Fig 1). The BTSC was designed to promote a cultural shift towards empowering patients and cultivated a supportive community through sharing of ideas via the SHAREHD website [31], newsletters and social media including #whyidosharedcare, Facebook, WhatsApp and @sharemydialysis on Twitter. An optional four-day nurse-led training course accredited by the Royal College of Nursing enabled a small number of kidney staff and patient representatives from each participating centre to learn practical aspects of shared HD care to take back to their centres [32]. This course covered learning styles, motivational interviewing, patient action-plan development, patient stories and research evidence of the benefits of self-management and has previously demonstrated positive staff attitude and culture change towards shared HD care and partnership working in attendees [32].

### Instrumentation

#### Patient questionnaire

The patient questionnaire was adapted from the existing UKRR ‘Your Health Survey’ (YHS) which combines demographic questions with several patient-reported outcome measures, including the validated Patient Outcome Scale Symptom list for patients with kidney failure (POS-S Renal) measuring symptom burden [33, 34], the validated EuroQol five Dimensions with five Levels questionnaire (EQ-5D-5L) measuring quality of life [35] and the PAM-13 measure of self-management ability. PAM-13 asks patients to rate the extent to which they agree with 13 statements about self-management using a Likert scale. PAM-13 results are reported as scores (0–100) and levels (1–4), where lower scores and levels indicate lower activation. Items of the PAM-13 can be found in the PAM-13 development paper [36]. The adapted patient questionnaire (YHS2) added questions on HHD interest, use of PatientView (a portal that allows patients to view their test results and clinical correspondence) and a validated single-item health literacy screening tool, derived from the Brief Health Literacy Score [37, 38]. These three questions were added as variables that may influence patient activation. Further information on how the above measures are scored is included in S1 Methods. To measure deprivation, patients’ postcodes were converted to Lower-layer Super Output Areas [39] and matched to adjusted UK Index of Multiple Deprivation scores and quintiles [40], enabling comparison between UK countries [41].

#### Staff questionnaire

The CSPAM questionnaire consists of 14 questions about clinicians’ attitudes towards self-management and nine questions about clinicians’ behaviours to support self-management [28]. 13 of the 14 attitude questions are combined to form a CSPAM score (0–100) and level (low, medium and high), where lower scores and levels indicate lower support for patient self-management [28]. Items of the CSPAM can be found in the CSPAM development paper [28].

### Statistical methods

The number of patient questionnaires we aimed to collect per centre was at least 50. This was determined by what was a realistic number to collect in the previous SHAREHD BTSC [27]. The number of staff questionnaires we aimed to collect per centre was at least 10. This was pragmatically determined by kidney centre staffing numbers and influenced by previous studies indicating that including any fewer than 10 CSPAM questionnaires limits generalisability [42].

Descriptive statistics were carried out for patient and staff data. Categorical patient/staff variables were recategorized based on similarity of scores with neighbouring groups if groups contained less than 10 individuals [43]. Continuous variables were recategorized based on their association with PAM or CSPAM score in a Loess smoothing plot. If a linear association was observed on a Loess smoothing plot, the variable was kept as a continuous variable. If step change(s) in PAM or CSPAM score were seen on a Loess smoothing plot, then the variable was converted into categories based on where the step change(s) in PAM or CSPAM score lay. For categorical variables, the category with the greatest number of patients or staff became the reference group in multivariable models. Mean (standard deviation [SD]) or medians (interquartile range [IQR]) were reported where data is normally distributed or skewed respectively. P<0.05 was considered statistically significant for all analyses. All analyses were conducted using SPSS version 25 and all statistical assumptions were tested.

#### Hypothesis one: Kidney staff CSPAM scores are significantly associated with patients’ PAM scores in their centre at baseline

Patients and staff were excluded from analyses involving PAM/CSPAM score if they had missed out at least one PAM/CSPAM question. Patients responding with all ‘agree strongly’ (score 100) or all ‘disagree strongly’ (score 0) were also excluded as were staff scoring 100 in the CSPAM at baseline. Such patient and staff responses are considered unreliable and result in measurement error [10, 28, 42, 44]. Insignia Health automatically assign patients who respond all ‘agree’ a Level 2, rather than Level 3, so as not to overestimate their PAM scores. Individuals responding with all ‘agree’ were additionally excluded in sensitivity analyses to determine whether their inclusion affected the results obtained.

Individual staff CSPAM scores and levels were combined with staff CSPAM responses from the same centre to give three variables: centre-level mean CSPAM score, centre-level proportion of staff scoring ‘high’ and centre-level proportion of staff scoring ‘low’. These three variables existed separately for all staff and for nurses and healthcare assistants (HCAs) only to form six centre-level variables in total. Nurses and HCAs spend more time with patients and may have a greater influence on patient activation. These six centre-level variables were used in univariate and multivariable analyses to match staff CSPAM scores to the PAM scores of patients at their kidney centre.

Univariate analyses predicting baseline patient-level PAM score were conducted with patient-level characteristics using the independent t-test, ANOVA, Mann-Whitney U and Kruskal Wallis tests, depending on the number of categories and skew. Patient-level variables included patient age, deprivation, symptoms, quality of life, health literacy, where and with whom PAM was completed with, HHD interest and use of PatientView. As anxiety and depression were asked about twice, together in the EQ-5D-5L and separately in the POS-S Renal, only the separate anxiety and depression variables of POS-S Renal were included in the multivariable model, if significant on univariate analyses, to avoid repetition and to study their individual effects on PAM score. Univariate analyses predicting baseline patient-level PAM scores were also carried out with the six centre-level CSPAM variables described above as predictors, through simple linear regression or chi-squared analysis depending on satisfaction of assumptions.

Patient-level variables that had a P value of <0.1 on univariate analyses with PAM score were included as potential confounders in multivariable linear regression models predicting baseline patient-level PAM score. Six main multivariable models were created, with each model using a different centre-level CSPAM variable as a predictor. Sensitivity analyses were conducted for all six main multivariable models, where patients answering all ‘agree’ were excluded, to investigate whether their inclusion in the final models affected the results. A clinically important difference in PAM score was defined as 4–5 points [45, 46].

#### Hypothesis two: The SHAREHD BTSC is associated with a significant increase in kidney staff CSPAM scores from baseline to six months

Univariate analyses predicting staff-level baseline CSPAM score were conducted with baseline staff-level variables, excluding staff scoring 100 at baseline, using the independent t-test, ANOVA, Mann-Whitney U and Kruskal Wallis tests, depending on the number of categories and skew. Staff-level variables consisted of age, gender, staff type and years of experience in chronic disease.

Depending on skew, paired samples t-tests or Wilcoxon signed-rank tests predicting staff-level six-month CSPAM scores were conducted with staff-level baseline CSPAM scores in the same individuals at both data collection periods. Staff scoring 100 at baseline were excluded, but those scoring 100 at six months were included to provide the greatest opportunity to observe a change in CSPAM score. Sensitivity analyses were then conducted which excluded staff scoring high-level activation at baseline, as these individuals may mask improvements in staff at low and medium levels.

Univariate analyses (independent t-test or Mann-Whitney U test) and multivariable linear regression analyses were used to predict staff-level six-month CSPAM scores with baseline CSPAM score or collection round as predictors in univariate and multivariable models respectively. Staff scoring 100 at baseline were excluded, while those scoring 100 at six months were included. Baseline staff-level variables that had a P value of <0.1 in univariate analyses predicting CSPAM score were included in a main multivariable model, alongside collection round, to predict CSPAM score. A sensitivity analysis included staff scoring 100 at baseline back into the multivariable model predicting staff-level six-month CSPAM score to test for the presence of a ceiling effect introduced by these individuals and to ensure that the SHAREHD BTSC did not reduce CSPAM scores. A ceiling effect would result in a smaller positive effect size in the sensitivity analysis than in the main final model, as individuals scoring 100 at baseline may also score highly at six months. Half the standard deviation of CSPAM score at baseline was used to define a clinically meaningful difference [47].

## Results

### Patient characteristics and association with PAM score

**Fig 2:**
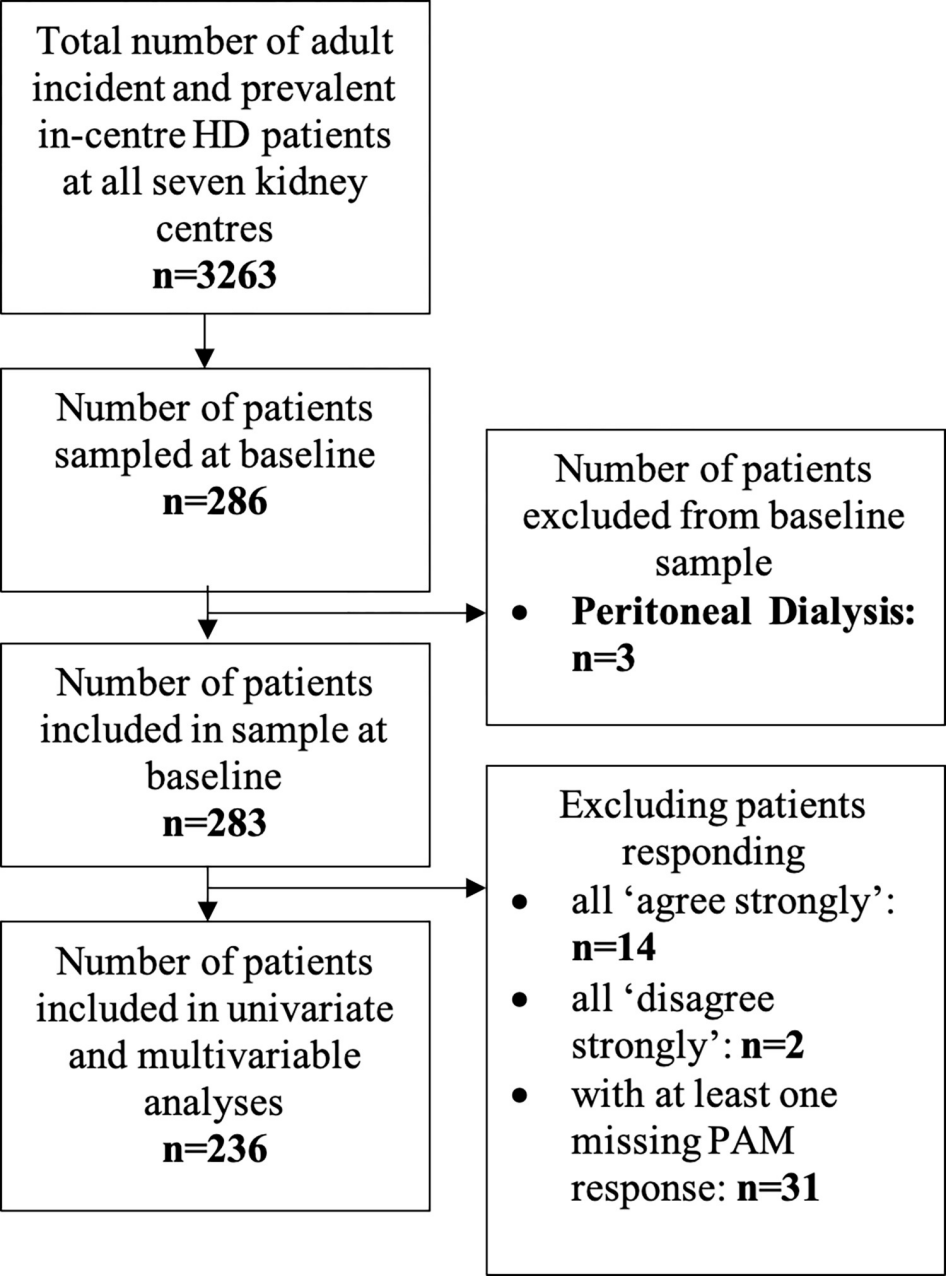
Flow diagram of patients included in analyses.

286 YHS2 baseline patient questionnaires were completed, of which three were for patients who received peritoneal dialysis and were excluded, leaving 283 patients (Fig 2 and Table 1). Median patient age was 63.0 years (range 19–91), and most completed the questionnaire in a clinical environment (78.8%) and on their own (53.4%) (S3 Table). 51.9% had adequate health literacy. 20.1% used ‘PatientView’ and 9.9% were interested in HHD. High symptom burden and difficulties with health-related quality of life were demonstrated (S3 and S4 Tables).

**Table 1:** Number (percentage) of patients and staff by kidney centre at baseline and six-month follow up.

		Number (% of study cohort from each kidney centre)		
Kidney centre	Total number of adult in-centre HD patients at each centre*	Baseline patient cohort	Baseline staff cohort	Follow up staff cohort
Belfast	246	40 (14.1)	12 (10.3)	11 (12.8)
Birmingham	1210	51 (18.0)	15 (12.8)	7 (8.1)
Crosshouse	179	26 (9.2)	10 (8.5)	12 (14.0)
James Cook	461	39 (13.8)	10 (8.5)	7 (8.1)
Liverpool	457	37 (13.1)	24 (20.5)	20 (23.3)
Salford	559	49 (17.3)	13 (11.1)	9 (10.5)
Western	151	41 (14.5)	33 (28.2)	20 (23.3)
**Total**	3263	283 (100)	117 (100)	86 (100)

*Data taken from UKRR 22nd Annual Report [48]

PAM score was normally distributed (range 0–100, mean 55.5) and generalisable to other studies involving CKD and HD patients [8, 14, 49]. Fig 2 shows the number of patients included in further analyses involving PAM. Patients were most frequently in PAM level 2 (39.8%), with fewest patients in PAM level 4 (12.7%) (S3 Table).

On univariate analysis, significantly higher PAM scores were obtained in patients under 50 years of age, those completing PAM with staff, with adequate health literacy, better quality of life and less severe itching, changes in skin, anxiety and depression (all P<0.05) (S3 Table). On multivariable analysis, only younger patient age, completion with staff, adequate health literacy and fewer problems with self-care remained statistically significant and clinically meaningful predictors of higher PAM scores (all P<0.02). Though PAM scores varied by deprivation quintile, there was no clear trend.

### Staff characteristics and association with baseline CSPAM score

**Fig 3:**
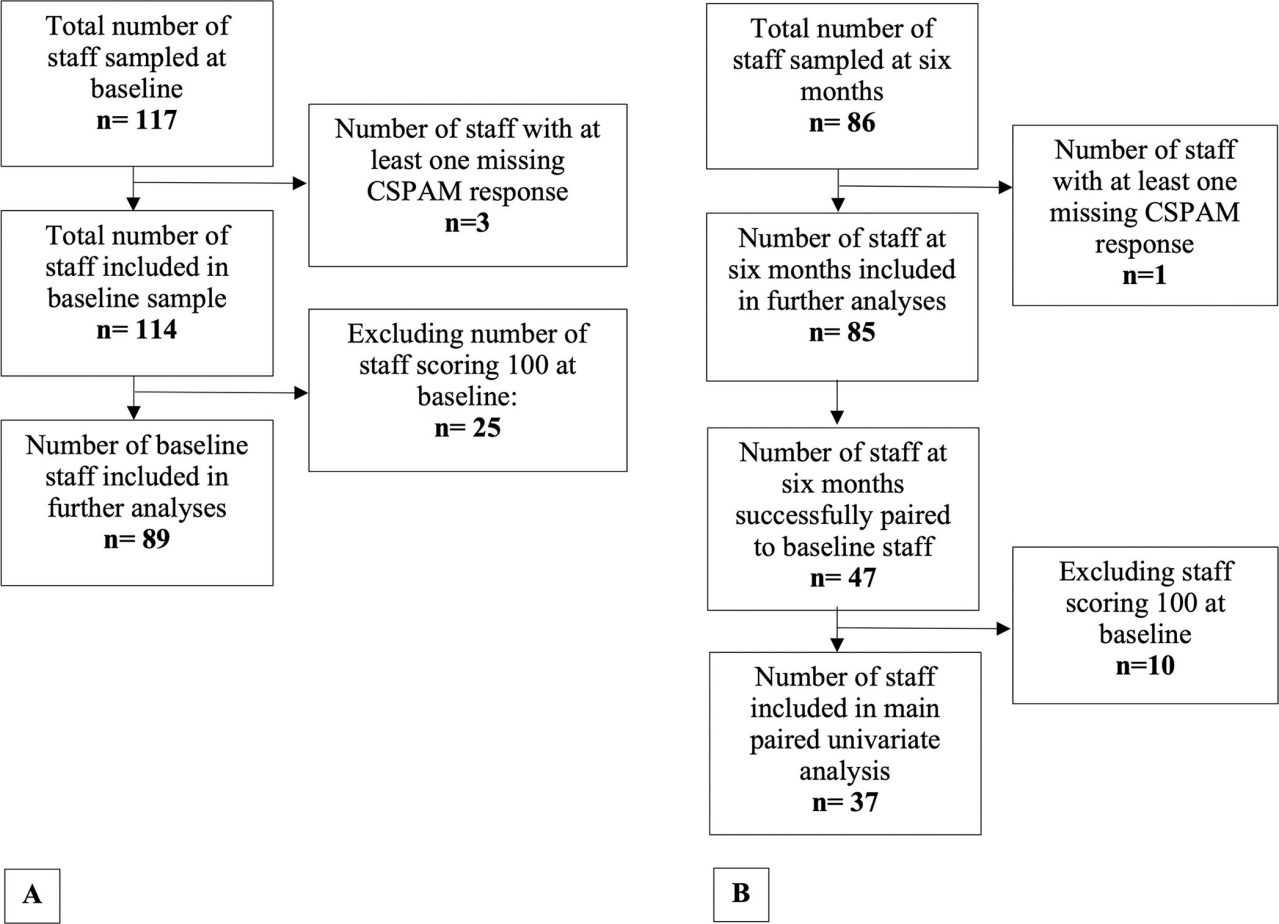
Flow diagram of staff included in analyses. (A) Inclusion in baseline staff sample and analyses; (B) inclusion in six-month staff sample and analyses.

There were more staff responses at baseline than at six months (Table 1). At both collection periods, staff were predominantly between 35–54 years, female and nursing staff (Table 2). Baseline CSPAM score was bimodally distributed (range 53.2–100, median 72.6) as was baseline mean centre-level CSPAM score. Fig 3A and 3B show staff numbers included in further analyses involving CSPAM. Staff were most frequently assigned a high CSPAM level at baseline (42.2%) (Table 2). In univariate analyses, only staff type was a significant predictor of CSPAM score (P<0.001). Younger and female staff tended to have higher CSPAM scores, though were non-significant (Table 2). There was no clear trend of CSPAM scores with years of experience.

**Table 2:** Staff-level characteristics, univariate analysis and main unpaired multivariable model predicting CSPAM score.

	Number (%)		Baseline univariate analysis (n = 89)		Main multivariable analysis (n = 174)‡ R^2^ = 0.137	
Staff-level characteristics	Baseline (n = 117)	Six months (n = 86)	Median (IQR)§	P value	Regression coefficient (95% CI)	P value
**Age**				0.345		
<35	24 (20.5)	18 (20.9)	72.6 (69.2 to 80.9)			
35–44	39 (33.3)	30 (34.9)	72.6 (64.3 to 78.7)			
45–54	42 (35.9)	29 (33.7)	72.6 (62.5 to 78.7)			
≥55	12 (10.3)	9 (10.5)	67.1 (62.6 to 71.4)			
Missing	0 (0)	0 (0)				
**Gender**				0.066		
Male	19 (16.2)	17 (19.8)	66.2 (60.8 to 75.4)		(Ref)	
Female	98 (83.8)	68 (79.1)	72.6 (66.1 to 78.7)		2.887 (-1.688 to 7.463)	0.215
Other	0 (0)	1 (1.2)	-		-	-
Missing	0 (0)	0 (0)				
**Staff type**				<0.001*		
Nurse	70 (59.8)	53 (61.6)	72.6 (66.1 to 78.7)		(Ref)	
HCA	17 (14.5)	14 (16.3)	78.7 (75.4 to 83.0)		1.653 (-3.051 to 6.357)	0.489
Doctor	21 (17.9)	14 (16.3)	67.1 (60.8 to 78.7)		-5.118 (-9.721 to -0.515)	0.030*
AHP and other†	9 (7.7)	5 (5.8)	61.7 (60.0 to 62.5)		-13.043 (-19.609 to -6.476)	<0.001*
Missing	0 (0)	0 (0)				
**Years of experience in chronic disease**				0.275		
0–5	22 (18.8)	15 (17.4)	72.6 (66.1 to 78.7)			
6–10	14 (12)	16 (18.6)	79.2 (65.3 to 83.0)			
11–15	33 (28.2)	19 (22.1)	68.1 (62.5 to 75.4)			
16–20	22 (18.8)	11 (12.8)	74.0 (66.1 to 78.7)			
>20	26 (22.2)	25 (29.1)	72.6 (60.8 to 78.7)			
Missing	0 (0)	0 (0)				
**CSPAM levels** ‡						
Low	25 (27.2)	24 (27.9)				
Medium	25 (27.2)	23 (26.7)				
High	39 (42.2)	38 (44.2)				
Missing	3 (3.3)	1 (1.2)				
**Collection round**						
Baseline					(Ref)	
Six months					1.339 (-1.945 to 4.623)	0.422

*P<0.05

†‘Other’ staff include dialysis assistants, student nurses, clinical support workers, ward clerks and patient dialysis engagement volunteers

‡Baseline CSPAM levels exclude staff scoring 100 on the CSPAM to leave n = 89 (excluding missing responses). Six-month CSPAM levels include staff scoring 100 to leave n = 85 (excluding missing responses). Total sample in multivariable analysis = 174 (89 +85).

§IQR = interquartile range

### Hypothesis one: Association between PAM and CSPAM at baseline

#### Univariate analyses

**Fig 4:**
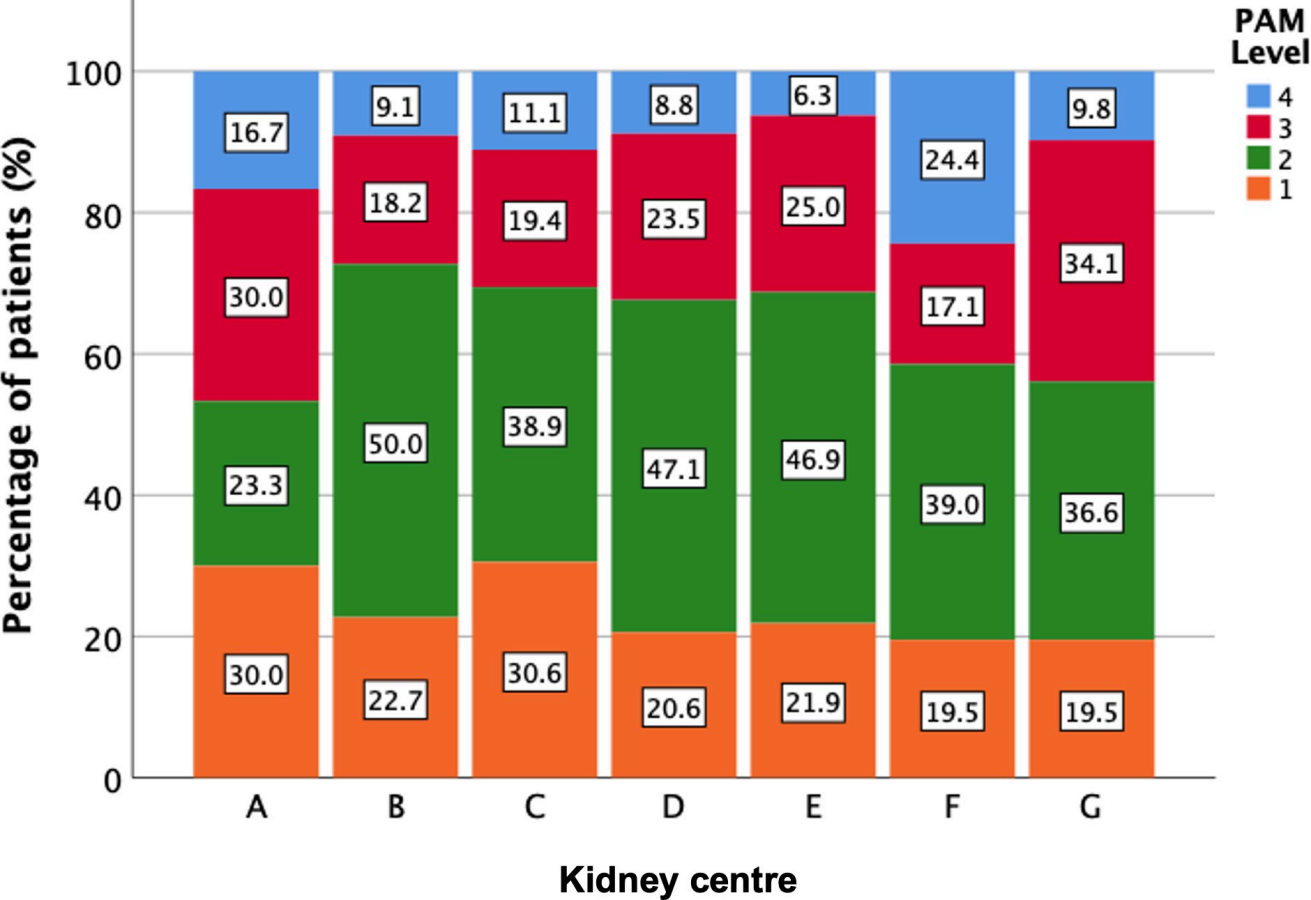
Stacked bar chart of PAM level by kidney centre. Kidney centres are anonymised using letters. Figures within stacks represent the percentage of patients within each level.

**Fig 5:**
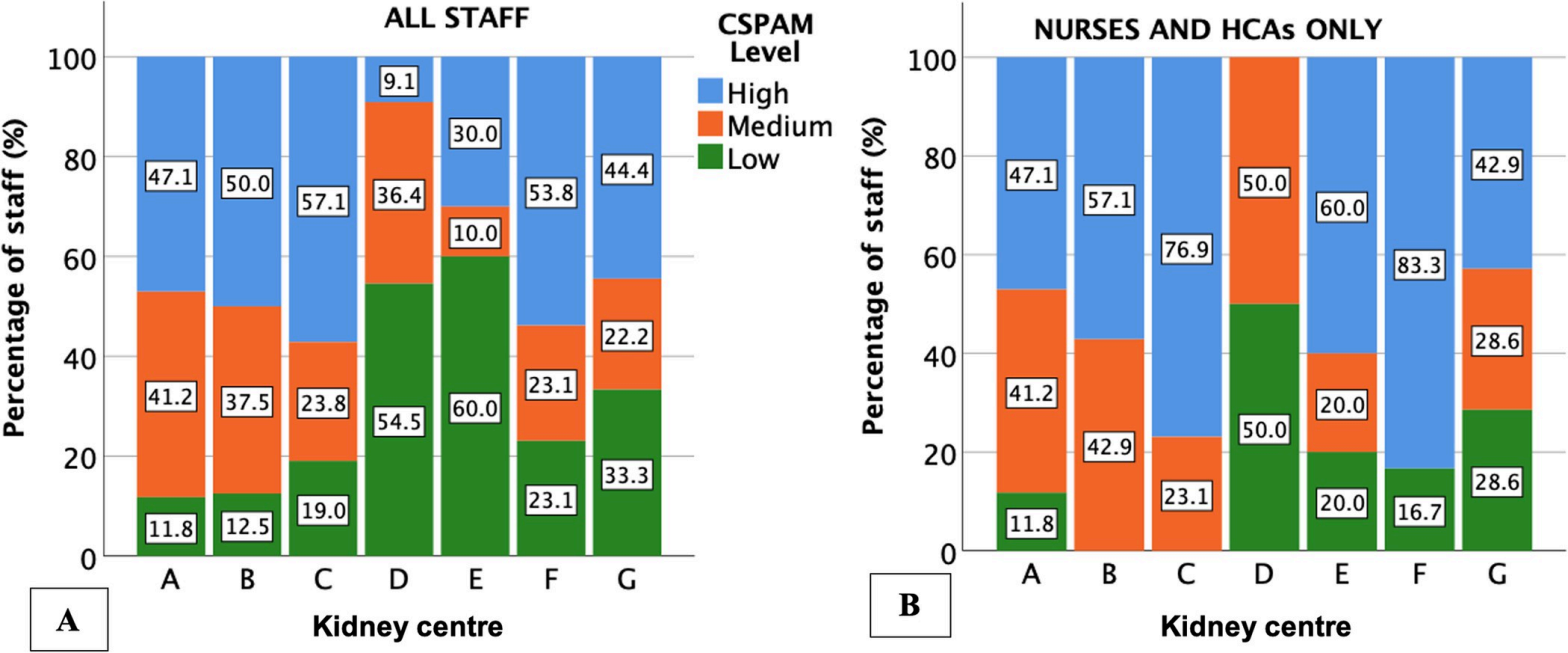
Stacked bar chart of baseline CSPAM level by kidney centre (anonymised using letters). Figures within stacks represent the percentage of staff within each level. (A) includes all staff types. (B) includes nurses and HCAs only.

**Fig 6:**
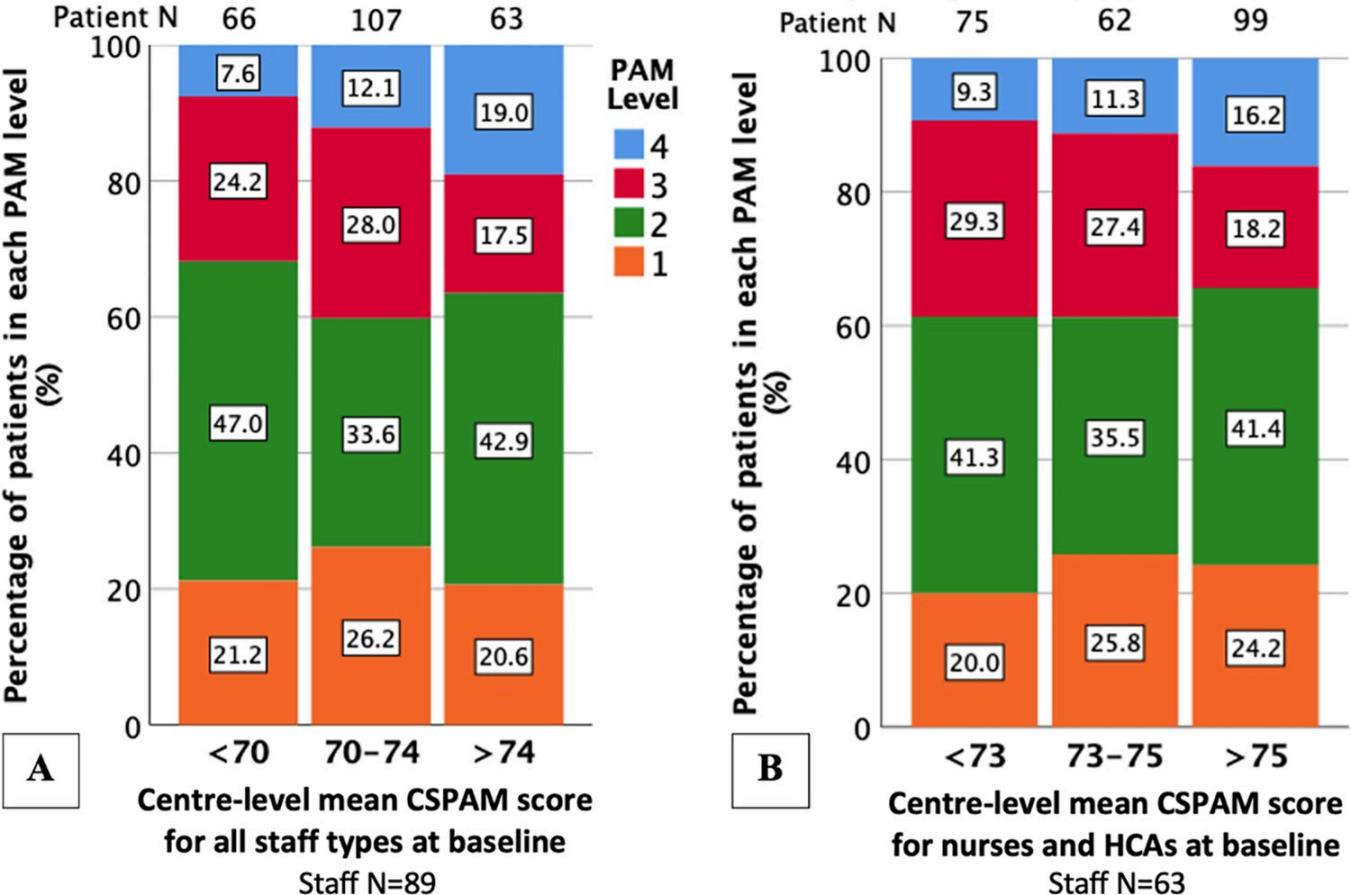
Stacked bar charts of categorised baseline centre-level mean CSPAM score for all staff and nurses and HCAs only by PAM level. Numbers within stacks represent the percentage of patients within each level. Patient N = number of patients represented by each column. (A) Chi-squared for all PAM levels: P = 0.321, Chi-squared for PAM level 4: P = 0.052. (B) Chi-squared for all PAM levels: P = 0.917, Chi-squared for PAM level 4: P = 0.174.

There was less variability in PAM levels by centre than in CSPAM levels by centre–Figs 4 and 5 respectively. The proportion of patients in each PAM level was similar across categories of baseline centre-level CSPAM (P = 0.321 for all staff and 0.917 for nurses and HCAs only)–Fig 6A and 6B. Results were similar when limiting the analysis to the proportion of patients in PAM level 4 only and when using baseline centre-level proportion of staff with high-level or low-level activation as opposed to mean centre-level CSPAM score—S1 and S2 Figs.

#### Multivariable analyses

In multivariable analysis, increasing baseline centre-level mean CSPAM score by one point for all staff resulted in a non-significant 0.3-point increase in PAM score (regression coefficient: 0.328 (95% CI: -0.157 to 0.812); P = 0.184) (Table 3). Increasing CSPAM score by a clinically meaningful number of points (4.75 CSPAM points) would therefore lead to a 1.4-point increase in PAM score, which is not clinically meaningful (4–5 PAM points) [45, 46]. Though the variables in the multivariable model explained 28.4% of the variance in PAM score, only 0.2% of this was due to baseline centre-level mean CSPAM score in all staff. Similar results were obtained for baseline centre-level mean CSPAM score for nurses and HCAs only (0.214 (95% CI: -0.185 to 0.614); P = 0.291) (Table 3). Likewise, baseline centre-level proportion of staff with high or low-level activation were not significantly associated with PAM score in separate models, regardless of staff type included (Table 3). Excluding individuals scoring all ‘agree’ to PAM questions in sensitivity analyses did not impact significantly on the results obtained (sensitivity analysis regression coefficient for centre-level mean CSPAM score for all staff: 0.375 (95% CI: -0.148 to 0.898); P = 0.159) (Table 3).

**Table 3:** Regression coefficients and P values of baseline centre-level CSPAM predictors in the main multivariable models and sensitivity analyses predicting PAM score.

	Main regression analyses (n = 197)*			Sensitivity analyses (n = 178)*		
Baseline centre-level CSPAM Predictors	Regression coefficient (95% CI)	P value	R^2^	Regression coefficient (95% CI)	P value	R^2^
All staff: mean CSPAM score	0.328 (-0.157 to 0.812)	0.184	0.284	0.375 (-0.148 to 0.898)	0.159	0.318
All staff: % high CSPAM level (per 10%)	0.656 (-0.330 to 1.643)	0.191	0.283	0.737 (-0.336 to 1.809)	0.177	0.317
All staff: % low CSPAM (per 10%)	-0.512 (-1.482 to 0.458)	0.299	0.281	-0.548 (-1.593 to 0.496)	0.301	0.314
Nurses and HCAs: mean CSPAM score	0.214 (-0.185 to 0.614)	0.291	0.281	0.217 (-0.218 to 0.652)	0.326	0.314
Nurses and HCAs: % high CSPAM level (per 10%)	0.280 (-0.286 to 0.846)	0.330	0.280	0.274 (-0.340 to 0.887)	0.380	0.313
Nurses and HCAs: % low CSPAM level (per 10%)	-0.480 (-1.535 to 0.576)	0.371	0.280	-0.445 (-1.600 to 0.709)	0.447	0.312

*n values = number of patients included in each analyses type

### Hypothesis two: Change in CSPAM score over six months

#### Univariate analyses

Range and median CSPAM scores were similar at baseline (range 53.2–100, median 72.6) and six months (range 51.7–100, median 72.6) with both baseline and six-month CSPAM scores being bimodally distributed. A clinically important difference in CSPAM score was determined to be 4.75 points. 25 staff scored 100 at baseline (21.9%) compared to nine staff (10.6%) at six months. After removing staff scoring 100 at baseline (Fig 3A), unpaired analysis showed median CSPAM scores were unchanged from baseline [72.6 (IQR = 64.3 to 78.7)] to six months [72.6 (IQR = 63.4 to 83.0; P = 0.662)]. In paired staff (Fig 3B), median CSPAM scores also remained the same at baseline [68.1 (IQR = 60.8 to 75.4)] and six months: [68.1 (IQR = 60.0 to 77.1); P = 0.896)]. Excluding staff scoring a high level at baseline in paired sensitivity analyses (leaving n = 26), showed similar results [baseline median CSPAM score: 64.3 (IQR = 60.8 to 68.6), six months median CSPAM score 64.3 (IQR = 57.2 to 70.8); P = 0.939)]. Staff-level characteristics were similar in both paired and unpaired analyses (S5 Table).

#### Multivariable analyses

On multivariable modelling (Table 2), doctors, AHPs and other staff showed significantly lower CSPAM scores compared to nurses that were potentially clinically meaningful. Female staff tended to have higher CSPAM scores, but this did not reach significance. There was still no significant difference between CSPAM scores at baseline and six months (1.339 (-1.945 to 4.623); P = 0.422). Though the variables in the multivariable model accounted for 13.7% of the variance in CSPAM score, only 0.8% of this was due to collection round. With the inclusion of the 25 staff scoring 100 at baseline in sensitivity analyses, CSPAM scores showed a statistically significant 4.5-point decrease from baseline to six months (-4.535 (-8.292 to -0.778); P = 0.018) and R^2^ = 0.134.

## Discussion

### Summary of findings

This multi-centre study was the first to examine the association between staff attitudes and patient self-management specifically within in-centre HD, as measured by the PAM and CSPAM, and to investigate whether staff training in the SHAREHD QI programme could lead to a modification of CSPAM scores after six months. Both univariate and multivariable analyses revealed a non-significant baseline association between PAM and CSPAM and a non-significant change in CSPAM scores that were not clinically meaningful. Lower PAM scores were associated with older age, more problems with symptoms and quality of life and limited health literacy. Lower CSPAM scores were seen in doctors, AHPs and ‘other’ category staff.

### Possible mechanisms and explanations

Discussion of relationships between patient-level characteristics and PAM score and staff-level characteristics and CSPAM score can be found in S1 Discussion.

#### Hypothesis one: Association between PAM and CSPAM

The lack of centre-level association between PAM and CSPAM was suggested by the greater variability in CSPAM levels than PAM levels by centre. Variation in CSPAM levels by centre may be due to differences in workplace culture, local resources, selection bias, fewer staff participants relative to patients and variation in the proportion of nurses and HCAs sampled by centre, who tend to have higher CSPAM scores than other staff [29, 42].

The lack of association between PAM and CSPAM may stem from limitations in the measures or an inherent disconnect between patient and staff attitudes. While the validity of the PAM has been tested in CKD, neither the validity of the PAM nor the CSPAM has been tested in the HD setting [50]. PAM is not condition-specific, so patients might respond considering their ability to manage other conditions not under the care of kidney clinicians [51]. Additionally, kidney failure necessitates specific self-management tasks that may warrant disease-specific questions [50]. Exercise-related questions may be difficult for some patients due to self-care issues or significant symptoms, rather than a lack of activation. Moreover, questions such as ’I know how to prevent problems with my health’ may be unsuitable for patients with incurable conditions like kidney failure [50, 51]. Some CSPAM questions appear too forceful or extreme, leading to a lack of clinician endorsement [42, 52]. One example asks staff how important it is for patients to carry out treatments they have been ‘told’ to do at home, which may sound paternalistic. ‘When all is said and done, they [the patient] are the ones responsible for managing their health’ is often perceived as inflexible. Staff state that patients are not alone and that care is a shared partnership [42], with levels of responsibility tailored to individual needs and preferences rather than a ‘one size fits all’ approach where patients are forced into directions they are not ready to take [42, 53]. This is especially as patient activation is a journey from PAM level 1 at diagnosis to PAM level 4, with differing staff input required at different parts of patient progression. Though a tailored self-management approach is supported by meta-analyses, such responses result in lower CSPAM scores [54, 55]. It may be particularly challenging for kidney clinicians to support patients on HD to ‘work out new solutions’ or ‘manage the problem on their own,’ because patients on HD are high-risk and dialysis treatment is very complex, with patients being older, frail, multi-morbid and experiencing cognitive impairment and dementia [42, 56]. HD patients may prefer to see their specialist team for advice, as general practitioners may not be confident in managing kidney impairment [57].

Alternatively, there might be no inherent connection between patient and staff attitudes towards self-management. Gair et al. [13] found no link between PAM and CSPAM in pre-dialysis and kidney replacement therapy patients. Alvarez et al. [58] observed a weak association (r = 0.28), but did not report a p value. Positive staff attitudes may not translate to patients due to organisational factors such as time constraints and short-staffing [14, 29]. The ’transaction hypothesis’ suggests that highly activated patients are more adept in gaining what they require from clinicians and healthcare systems, as more highly activated patients report better healthcare experiences than those with lower activation, despite being under the care of the same clinician [11]. This suggests that patients themselves may have more influence over their self-management than staff attitudes.

#### Hypothesis two: Change in CSPAM post-intervention

The CSPAM is derived from PAM items [28]. While PAM items can show a ceiling effect [50], sensitivity of the CSPAM to change has not been explored to our knowledge. The aforementioned limitations and generic nature of the CSPAM combined with persistent organizational barriers may have hindered staff from improving their baseline responses in paired and main unpaired analyses [29]. The unpaired sensitivity analysis showed a reduction in scores when staff scoring 100 at baseline were included. Staff understanding of person-centred care may have increased, causing staff to be more critical at six months. This recalibration of ideas and attitudes is known as a ‘response shift’ [59] and occurs after educational interventions, where participants overestimate their capabilities at baseline [60]. As a result of this overall decrease in scores, a ceiling effect in the CSPAM was not detected, but could not be excluded.

Repeating our study with the same methods after the COVID-19 pandemic is unlikely to change our results for both hypotheses, as the effect sizes were small and non-significant. Though COVID-19 has increased time pressures, which may make it more challenging for clinicians to support patient activation [29], our experience was that staff still had positive attitudes towards shared HD care, but prioritisation of patient flow was a challenge to its maintenance at pre-COVID levels. This led to centres relaunching their shared HD care programmes to regenerate interest when infection rates lessened. Factors promoting the maintenance of shared HD care despite COVID-19 included senior buy-in, co-production, peer support and dedicated staff champions [22]. The difference in COVID-19 infection rates between in-centre HD and HHD patients highlights the importance of initiatives to promote self-management [61] and our team has developed an effective, virtual version of the nurse-led shared HD care training course [22].

### Strengths and limitations

Strengths of this study include its multicentre nature, specific context of in-centre HD, multivariable adjustment and generalisability of kidney staff age and gender and patient age, quality of life and symptom burden to other studies involving pre-dialysis patients and those receiving kidney replacement therapy [1, 14, 42]. Possible selection bias could have been present, and its direction may have varied by centre. Some staff champions might have chosen staff they believed would be more supportive of patient self-management, while others might have aimed for a wider range of views. Although this study had a higher proportion of staff with high-level CSPAM scores (baseline: 42.2% [excluding staff scoring 100]; six months: 44.2%) compared to another study involving kidney staff (30%), median CSPAM scores were similar (72.6 versus 71.6 respectively) [42]. Despite potential selection bias, there was still no significant change in paired sensitivity analyses that excluded staff with high scores at baseline. YHS2 was only available in English, which could have restricted participation. Reasons for non-participation by patients and staff were not collected, meaning biases in non-participants were not assessed. Formal power calculations were not carried out, and staff sickness and turnover led to fewer than 10 staff responses from three centres at six months. These factors, along with difficulty in reliably pairing some baseline and follow-up responses due to similar age, gender, and initials, resulted in small numbers in paired analyses. Unpaired samples meant that new staff were introduced at six months that may not have been exposed to shared HD care for as long. This could have limited the ability to detect a change in CSPAM after completion of the BTSC. A disease-specific questionnaire measuring staff attitudes to shared HD care may have yielded different results. A larger staff sample may have narrowed the confidence intervals but given the small effect size obtained in unpaired multivariable analyses (regression coefficient = 1.238), it is unlikely that the change in CSPAM score after six months would become clinically meaningful. Patient vintage and the length of time staff had been working at each centre were not collected, meaning staff exposure time was not assessed as a potential effect modifier. However, the staff sampled provide an indication of the culture and organisational barriers at the centre, even if they may not have had prolonged contact with all the sampled patients. The fact that HD staff and patients frequently move around and may not consistently be in contact with each other may inherently contribute to why CSPAM does not correlate with PAM, as the workplace environment is very complex and many other factors influence patient activation.

### Policy and practice implications

This study’s findings imply that addressing staff attitudes alone is unlikely to significantly improve patient self-management. This is supported by a Cochrane review of 43 trials suggesting that condition-specific person-centred interventions involving both patients and clinicians, rather than clinicians alone, are more effective in enhancing patient satisfaction and health behaviours [62]. NHS England recommends a three-pronged approach to patient activation: patient-level, clinician-level, and organizational-level interventions. This aligns with the House of Care model for long-term conditions and a recent literature review on patient activation in CKD [6, 63].

At the patient level, self-management programmes have already been shown to be effective at improving self-efficacy, physiological markers, depression and patient activation, especially those that are individualised and culturally tailored, interactive, multifaceted and co-produced [54, 55, 64–67]. Behavioural change strategies are most effective and include health coaching tailored to PAM level, motivational interviewing, problem solving, goal setting and care plan development [55, 65, 68–70]. Face-to-face and remote programmes may be equally effective—a positive finding in the post-COVID era [54]. However, there is a paucity of studies using PAM as an outcome measure of HD self-management interventions. Peer support is also associated with increased knowledge, self-efficacy, reassurance, control, acceptance and patient activation [71–75] and should be encouraged, while addressing mentor coaching and lack of staff training in peer support implementation, optimisation, and referral [76].

Increasing patient activation (mindset) without health literacy (skill set) may widen health inequalities, as those with lower health literacy may find it harder to implement changes effectively [77–79]. Health literacy can be approached as an ‘asset’ to be improved by improving patient self-efficacy, empowerment, healthcare navigation, critical analysis and social skills [80]. Such interventions include SHAREHD and interventions to improve patient negotiation and question-asking skills in consultations, the latter of which has shown clinically meaningful increases in PAM scores, especially in those with low baseline PAM scores [81, 82]. Health literacy can also be treated as a ‘risk factor’ to be mitigated by screening with Patient Reported Outcome Measures and tailoring written materials and verbal communication to literacy levels [83]. A meta-analysis of this approach found improved knowledge and confidence of disadvantaged patients and shared decision-making ability [84]. A public health approach to health literacy should also be taken [85].

At the clinician level, staff training should be used to assist the delivery of patient interventions and may influence healthcare culture and attitudes. This is especially as staff report lack of confidence in communication skills, person-centred methods and how to implement and refer for patient support [29, 76]. At the organisational level, longer appointment times, staff shortages and limited support services should be addressed [14, 29]. Easier referral systems and dedicated staff champions help improve patient uptake of self-management support [76].

### Future research

Further research should be conducted to identify cost-effective, tailored, interactive, co-produced, dialysis-specific interventions that have positive long-term effects on in-centre HD patient self-management and outcomes and ensure those less likely to participate are included. Effective condition-specific staff training in patient self-management and delivery of interventions, in the presence of workforce pressures, should be undertaken to ensure sustainability. Disease-specific measures of HD patient self-management and HD staff attitudes and skill acquisition should be developed and validated and their sensitivity to change assessed. Whether to continue to use the PAM and the CSPAM as outcome measures in disease-specific interventions needs to be weighed up in light of their limitations in the phrasing of items and their genericity. Future studies should assess the validity of the PAM and CSPAM in the HD setting and their association with HD-specific outcomes and sensitivity to change.

## Conclusions

Despite the opportunity to support patient self-management, no significant association between in-centre HD patient activation and clinician support for patient activation were observed. Staff attitudes did not improve significantly after the BTSC. This suggests that modifying staff beliefs alone is less likely to influence patient self-management. Co-produced interventions involving both patients and staff need to be designed, tested and delivered locally to support patient self-management.

## Supporting information

S1 Checklist *PLOS ONE* clinical studies checklist. (DOCX)

S2 Checklist STROBE Statement—checklist of items that should be included in reports of observational studies. (DOCX)

S1 Methods Further information on patient questionnaire scoring. (PDF)

S1 Discussion Discussion of findings for PAM score with patient-level characteristics and CSPAM score with staff-level characteristics. (PDF)

S1 Table Shared HD care tasks. (PDF)

S2 Table Three learning events forming the basis of the BTSC. (PDF)

S3 Table Patient characteristics, univariate analyses and final multivariable model predicting PAM score. (PDF)

S4 Table Symptoms with P>0.1 on univariate analysis predicting PAM score. (PDF)

S5 Table Comparison of staff-level characteristics in paired and unpaired univariate analyses. (PDF)

S1 Fig Stacked bar charts of categorised baseline centre-level percentage of staff with a high CSPAM level for all staff and nurses and HCAs only by PAM level. Figures within stacks represent the percentage of patients within each level. Patient N = number of patients represented in each column. (A) Chi-squared for all PAM levels: P = 0.478, Chi-squared for PAM level 4: P = 0.056. (B) Chi-squared for all PAM levels: P = 0.901, Chi-squared for PAM level 4: P = 0.101.
(PDF)

S2 Fig Stacked bar charts of categorised baseline centre-level percentage of staff with a low CSPAM level for all staff and nurses and HCAs only by PAM level. Figures within stacks represent the percentage of patients within each level. Patient N = number of patients represented in each column. (A) Chi-squared for all PAM levels: P = 0.947, Chi-squared for PAM level 4: P = 0.436. (B) Chi-squared for all PAM levels: P = 0.543, Chi-squared for PAM level 4: P = 0.581.
(PDF)
