# Supplementary material for: Supporting patient self-management: A cross-sectional and prospective cohort study investigating Patient Activation Measure (PAM) and Clinician Support for PAM scores as part of a multi-centre haemodialysis breakthrough series collaborative
Source: PLoS One. 2024 May 22;19(5):e0303299. doi: 10.1371/journal.pone.0303299 (PMC11111028; doi:10.1371/journal.pone.0303299)
Supplement: S2 Checklist — (DOCX) [file pone.0303299.s002.docx]

STROBE Statement—checklist of items that should be included in reports of observational studies

|  | Item No. | Recommendation | Page  No. | Relevant text from manuscript |
| --- | --- | --- | --- | --- |
| **Title and abstract** | 1 | (*a*) Indicate the study’s design with a commonly used term in the title or the abstract | 0 (title) | ‘cross-sectional and prospective cohort study’ |
|  |  | (*b*) Provide in the abstract an informative and balanced summary of what was done and what was found | 1-2 | Please see abstract. |
| Introduction | | | |  |
| Background/rationale | 2 | Explain the scientific background and rationale for the investigation being reported | 2-5 | Please see introduction. |
| Objectives | 3 | State specific objectives, including any prespecified hypotheses | 4-5 | In this stand-alone six-month SHAREHD BTSC, we first collected baseline cross-sectional in-centre HD patient PAM and HD staff CSPAM data from seven participating UK centres, hypothesising that staff attitudes towards self-management may be associated with their HD patients’ ability to self-manage at their centre. This is because higher CSPAM scores are associated with positive staff behaviours that may influence patient self-management ability [28, 29]. Secondly, we collected staff CSPAM data from the same participating centres post-intervention (at six months), hypothesising that longitudinal HD staff attitudes towards self-management may change by participating in the empowering and supportive shared HD care movement and seeing first-hand the beneficial patient impact that can be made within their own resource-tight settings, potentially eroding long held beliefs on care delivery [22]. If HD staff attitudes influence HD patient self-management and such staff attitudes can be modified, the case for implementing staff coaching to support patient self-management is strengthened. |
| Methods | | | |  |
| Study design | 4 | Present key elements of study design early in the paper | 5 | This study was conducted across seven UK kidney centres and collected cross-sectional, baseline in-centre HD patient data, including PAM, and longitudinal HD staff data, including CSPAM, at baseline and after a stand-alone six-month BTSC, known as SHAREHD. |
| Setting | 5 | Describe the setting, locations, and relevant dates, including periods of recruitment, exposure, follow-up, and data collection | 6-7 | See ‘Study setting and participants’ subsection |
| Participants | 6 | (*a*) *Cohort study*—Give the eligibility criteria, and the sources and methods of selection of participants. Describe methods of follow-up’  *Case-control study*—Give the eligibility criteria, and the sources and methods of case ascertainment and control selection. Give the rationale for the choice of cases and controls  *Cross-sectional study*—Give the eligibility criteria, and the sources and methods of selection of participants | 6-7 | Pages 6-7: Adult incident and prevalent in-centre HD patients were recruited into the study between April and May 2018 through completing the patient questionnaire. Patients could complete the questionnaire on their own or with assistance to increase participation in groups that may not otherwise have been represented, such as those with reduced health literacy or any individual need. Staff champions also agreed to circulate at least 10 paper CSPAM questionnaires to kidney staff with a variety of roles in their centre. By implementing shared HD care, we hypothesised that the workplace culture would change and therefore staff were sampled from the participating centres and not just from staff who attended the learning events. Kidney staff were recruited into the study at baseline (June - September 2018) and post-intervention six months later (December 2018 - March 2019) by completion of the staff CSPAM questionnaire. Sampling the same staff at both time periods was advised to enable comparison.    Information describing methods of follow-up (specific to staff participants only)  Page 7: On each page of the CSPAM questionnaires, a participant identification number consisting of renal centre number, respondent initials and collection round was used to identify the same individuals at first and second data collection. Staff champions were provided with a list of initials and job titles of baseline participating staff in their centre for repeat participation at six months. |
|  |  | (*b*) *Cohort study*—For matched studies, give matching criteria and number of exposed and unexposed  *Case-control study*—For matched studies, give matching criteria and the number of controls per case |  |  |
| Variables | 7 | Clearly define all outcomes, exposures, predictors, potential confounders, and effect modifiers. Give diagnostic criteria, if applicable | 11-15 | Page 11: PAM-13 results are reported as scores (0-100) and levels (1-4), where lower scores and levels indicate lower activation.  Page 14: Individual staff CSPAM scores and levels were combined with staff CSPAM responses from the same centre to give three variables: centre-level mean CSPAM score, centre-level proportion of staff scoring ‘high’ and centre-level proportion of staff scoring ‘low’. These three variables existed separately for all staff and for nurses and healthcare assistants (HCAs) only to form six centre-level variables in total.  Page 14: Patient-level variables included patient age, deprivation, symptoms, quality of life, health literacy, where and with whom PAM was completed with, HHD interest and use of PatientView.  Page 15: Staff-level variables consisted of age, gender, staff type and years of experience in chronic disease. |
| Data sources/ measurement | 8* | For each variable of interest, give sources of data and details of methods of assessment (measurement). Describe comparability of assessment methods if there is more than one group | 11-12, S1 Methods | See ‘Instrumentation’ section. |
| Bias | 9 | Describe any efforts to address potential sources of bias | 6, 15 | Page 6: Patients could complete the questionnaire on their own or with assistance to increase participation in groups that may not otherwise have been represented, such as those with reduced health literacy or any individual need.  Page 15: Describes paired univariate and unpaired multivariable analyses performed to address small numbers in paired analyses |
| Study size | 10 | Explain how the study size was arrived at | 12 | The number of patient questionnaires we aimed to collect per centre was at least 50. This was determined by what was a realistic number to collect in the previous SHAREHD BTSC [27]. The number of staff questionnaires we aimed to collect per centre was at least 10. This was pragmatically determined by kidney centre staffing numbers and influenced by previous studies indicating that including any fewer than 10 CSPAM questionnaires limits generalisability [42]. |

| Quantitative variables | 11 | Explain how quantitative variables were handled in the analyses. If applicable, describe which groupings were chosen and why | 13 | Categorical patient/staff variables were recategorized based on similarity of scores with neighbouring groups if groups contained less than 10 individuals [43]. Continuous variables were recategorized based on their association with PAM or CSPAM score in a Loess smoothing plot. If a linear association was observed on a Loess smoothing plot, the variable was kept as a continuous variable. If step change(s) in PAM or CSPAM score were seen on a Loess smoothing plot, then the variable was converted into categories based on where the step change(s) in PAM or CSPAM score lay. For categorical variables, the category with the greatest number of patients or staff became the reference group in multivariable models. |
| --- | --- | --- | --- | --- |
| Statistical methods | 12 | (*a*) Describe all statistical methods, including those used to control for confounding | 12-16 | See ‘Statistical methods’ section. |
|  |  | (*b*) Describe any methods used to examine subgroups and interactions | 15-16 | See section 12*(e)* sensitivity analyses |
|  |  | (*c*) Explain how missing data were addressed | 13, 16 | Page 13: Patients and staff were excluded from analyses involving PAM/CSPAM score if they had missed out at least one PAM/CSPAM question. Patients responding with all ‘agree strongly’ (score 100) or all ‘disagree strongly’ (score 0) were also excluded as were staff scoring 100 in the CSPAM at baseline. Such patient and staff responses are considered unreliable and result in measurement error [10, 28, 42, 44]. Insignia Health automatically assign patients who respond all ‘agree’ a Level 2, rather than Level 3, so as not to overestimate their PAM scores. Individuals responding with all ‘agree’ were additionally excluded in sensitivity analyses to determine whether their inclusion affected the results obtained.  Page 16: A sensitivity analysis included staff scoring 100 at baseline back into the multivariable model predicting staff-level six-month CSPAM score to test for the presence of a ceiling effect introduced by these individuals and to ensure that the SHAREHD BTSC did not reduce CSPAM scores. |
|  |  | (*d*) *Cohort study*—If applicable, explain how loss to follow-up was addressed  *Case-control study*—If applicable, explain how matching of cases and controls was addressed  *Cross-sectional study*—If applicable, describe analytical methods taking account of sampling strategy | 24 | Use of paired and unpaired staff analyses as comparison. |
|  |  | (*e*) Describe any sensitivity analyses | 15-16 | Page 15: Sensitivity analysis of final multivariable models predicting PAM score (hypothesis 1):  Sensitivity analyses were conducted for all six main multivariable models, where patients answering all ‘agree’ were excluded, to investigate whether their inclusion in the final models affected the results. A clinically important difference in PAM score was defined as 4-5 points [45, 46].  Page 15: Sensitivity analysis of paired univariate analysis predicting CSPAM score at six months: (hypothesis 2)  Sensitivity analyses were then conducted which excluded staff scoring high-level activation at baseline, as these individuals may mask improvements in staff at low and medium levels.  Page 16: Sensitivity analysis of final multivariable model predicting CSPAM score (hypothesis 2):  A sensitivity analysis included staff scoring 100 at baseline back into the multivariable model predicting staff-level six-month CSPAM score to test for the presence of a ceiling effect introduced by these individuals and to ensure that the SHAREHD BTSC did not reduce CSPAM scores. A ceiling effect would result in a smaller positive effect size in the sensitivity analysis than in the main final model, as individuals scoring 100 at baseline may also score highly at six months. |
| Results | | | | |
| Participants | 13* | (a) Report numbers of individuals at each stage of study—eg numbers potentially eligible, examined for eligibility, confirmed eligible, included in the study, completing follow-up, and analysed | 16-18 | Table 1 and Figs 2 and 3 |
|  |  | (b) Give reasons for non-participation at each stage | 28 | ‘Strengths and limitations’ section  staff sickness and turnover led to fewer than 10 staff responses from three centres at six months.  Reasons for non-participation in patients was not collected and is acknowledged as a limitation. |
|  |  | (c) Consider use of a flow diagram | 16, 18 | Figs 2 and 3 are both flow diagrams |
| Descriptive data | 14* | (a) Give characteristics of study participants (eg demographic, clinical, social) and information on exposures and potential confounders | 16-19 | 16-17: patient descriptive data including S3 table  17-19: staff descriptive data including Table 2 |
|  |  | (b) Indicate number of participants with missing data for each variable of interest | 16  (S3 Table)  19-20 (Table 2) | S3 Table and Table 2 |
|  |  | (c) *Cohort study*—Summarise follow-up time (eg, average and total amount) | 7 | Kidney staff were recruited into the study at baseline (June - September 2018) and post-intervention six months later (December 2018 - March 2019) by completion of the staff CSPAM questionnaire. |
| Outcome data | 15* | *Cohort study*—Report numbers of outcome events or summary measures over time | 19-20 | Table 2 – staff data was collected as part of prospective cohort study design |
|  |  | *Case-control study—*Report numbers in each exposure category, or summary measures of exposure |  |  |
|  |  | *Cross-sectional study—*Report numbers of outcome events or summary measures | 16 | S3 Table – baseline patient data was cross-sectional |
| Main results | 16 | (*a*) Give unadjusted estimates and, if applicable, confounder-adjusted estimates and their precision (eg, 95% confidence interval). Make clear which confounders were adjusted for and why they were included | 16  (S3 Table)  19-20 (Table 2) | S3 Table and Table 2 |
|  |  | (*b*) Report category boundaries when continuous variables were categorized | 16  (S3 Table) | S3 Table (patient age was a continuous variable that was recategorized) |
|  |  | (*c*) If relevant, consider translating estimates of relative risk into absolute risk for a meaningful time period | N/A | N/A |

| Other analyses | 17 | Report other analyses done—eg analyses of subgroups and interactions, and sensitivity analyses | 22, 24-25 | Page 22: Excluding individuals scoring all ‘agree’ to PAM questions in sensitivity analyses did not impact significantly on the results obtained (sensitivity analysis regression coefficient for centre-level mean CSPAM score for all staff: 0.375 (95% CI: -0.148 to 0.898); P=0.159) (S4 Table).  Page 24: Excluding staff scoring a high level at baseline in paired sensitivity analyses (leaving n=26), showed similar results [baseline median CSPAM score: 64.3 (IQR=60.8 to 68.6), six months median CSPAM score 64.3 (IQR=57.2 to 70.8); Wilcoxon signed-rank test: P=0.939)].  Page 24-25: With the inclusion of the 25 staff scoring 100 at baseline in sensitivity analyses, CSPAM scores showed a statistically significant 4.5-point decrease from baseline to six months (-4.535 (-8.292 to -0.778); P=0.018) and R^2^=0.134. |
| --- | --- | --- | --- | --- |
| Discussion | | | | |
| Key results | 18 | Summarise key results with reference to study objectives | 25 | See ‘Summary of findings’ paragraph in discussion |
| Limitations | 19 | Discuss limitations of the study, taking into account sources of potential bias or imprecision. Discuss both direction and magnitude of any potential bias | 28-29 | See ‘Strengths and limitations’ paragraph of discussion |
| Interpretation | 20 | Give a cautious overall interpretation of results considering objectives, limitations, multiplicity of analyses, results from similar studies, and other relevant evidence | 25-28,  30-31 | Page 25-28: ‘Possible mechanisms and explanations’ section  Page 30-31: ‘Policy and practice implications’ section |
| Generalisability | 21 | Discuss the generalisability (external validity) of the study results | 28 | Strengths of this study include its multicentre nature, specific context of in-centre HD, multivariable adjustment and generalisability of kidney staff age and gender and patient age, quality of life and symptom burden to other studies involving pre-dialysis patients and those receiving kidney replacement therapy [1, 14, 42]. |
| Other information | |  | | |
| Funding | 22 | Give the source of funding and the role of the funders for the present study and, if applicable, for the original study on which the present article is based | Submission form | This study was funded by the Scaling Up Improvement Award from The Health Foundation awarded to Sheffield Teaching Hospitals NHS Foundation Trust (award number: 7664). |

*Give information separately for cases and controls in case-control studies and, if applicable, for exposed and unexposed groups in cohort and cross-sectional studies.

**Note:** An Explanation and Elaboration article discusses each checklist item and gives methodological background and published examples of transparent reporting. The STROBE checklist is best used in conjunction with this article (freely available on the Web sites of PLoS Medicine at http://www.plosmedicine.org/, Annals of Internal Medicine at http://www.annals.org/, and Epidemiology at http://www.epidem.com/). Information on the STROBE Initiative is available at www.strobe-statement.org.
