# Supplementary material for: Supporting patient self-management: A cross-sectional and prospective cohort study investigating Patient Activation Measure (PAM) and Clinician Support for PAM scores as part of a multi-centre haemodialysis breakthrough series collaborative
Source: PLoS One. 2024 May 22;19(5):e0303299. doi: 10.1371/journal.pone.0303299 (PMC11111028; doi:10.1371/journal.pone.0303299)
Supplement: S1 Methods — (PDF) [file pone.0303299.s003.pdf]

S1 Methods. Further information on patient questionnaire scoring.

The Patient Outcome Scale Symptom list for patients with kidney failure (POS-S Renal) asks to what extent patients have been affected by 17 kidney failure symptoms over the past week (not at all, slightly, moderately, severely, overwhelmingly, scored from 0-4 respectively). The EuroQol five Dimensions with five Levels questionnaire (EQ-5D-5L) assesses difficulty with mobility, self-care, usual activities, pain/discomfort and anxiety/depression (none, slight, moderate, severe and overwhelming). The single-item health literacy screening tool asked patients how confident they were at filling out medical forms by themselves (scores of ‘not at all,’ ‘a little bit’ and ‘somewhat’ = limited health literacy; scores of ‘quite a bit’ or ‘extremely confident’ = adequate health literacy).
