# Supplementary material for: Supporting patient self-management: A cross-sectional and prospective cohort study investigating Patient Activation Measure (PAM) and Clinician Support for PAM scores as part of a multi-centre haemodialysis breakthrough series collaborative
Source: PLoS One. 2024 May 22;19(5):e0303299. doi: 10.1371/journal.pone.0303299 (PMC11111028; doi:10.1371/journal.pone.0303299)
Supplement: S1 Discussion — (PDF) [file pone.0303299.s004.pdf]

S1 Discussion. Discussion of findings for PAM score with patient-level characteristics and CSPAM score with staff-level characteristics.

PAM and patient-level characteristics

Average PAM scores (mean=55.5, median=53.2) were similar to the Transforming Participation in CKD (TP-CKD) study (median=53.0) [1]. Higher proportion in PAM levels 1 and 2 (63.1%) vs. the TP-CKD study (51.6%) is likely due to the variety of kidney patient groups sampled in TPCKD, who have higher activation than in-centre HD patients [2]. Our cohort had lower average patient activation scores compared to studies in other chronic conditions (range: 59.4-66.2) [3-6], while noting that PAM scores of 100 were excluded in our study for reliability.

Other studies have also found lower patient activation in older kidney patients [2, 7-11]. Previous authors suggest limited health literacy [6, 10], depression, cognitive impairment [12] and functional difficulties [8, 9], may be contributing. However, older age remained statistically significant despite adjustment for these factors in our study and others [6, 10]. Polypharmacy, higher comorbidity and traditional hierarchical healthcare beliefs may be responsible [6, 8, 11, 13].

Symptoms and lower quality of life are known predictors of lower PAM scores in kidney patients [2, 7, 9, 10, 14, 15]. Effective self-management may alleviate symptom burden, while high symptom burden could hinder self-management. This emphasizes the importance of gathering Patient Reported Outcome Measures and tailoring symptom management to enhance activation [15]. Our finding of itching's impact on PAM scores is supported by a national

Canadian study where it was identified as the second most important research priority by patients with end-stage kidney disease [16]. After adjustment, only self-care remained a significant predictor of PAM score from the symptom and quality of life measures. Patients facing self-care challenges may also encounter issues with mobility, mental health, and experience a higher symptom burden, necessitating targeted support for self-management.

This study and others highlight a significant association between health literacy or educational level and patient activation in HD patients [11, 14, 17] and other populations [18-20]. Limited health literacy correlates with fewer self-care behaviours [21], overwhelm [22], passivity in shared decision making [23, 24], missed dialysis sessions and increased emergency department visits and hospitalisations [25]. While some studies outside the HD context show mixed results [26-28], our findings emphasize the need to address health literacy to enhance patient activation.

Deprivation quintiles showed no consistent trend with patient activation in this study. Similar findings in kidney patient studies may be due to the inconsistent relationship between postcode and deprivation [2, 9]. A systematic review indicates patients with higher socioeconomic status tend to seek active involvement, in contrast to more deprived patients who often prefer traditional healthcare roles [13]. Therefore, less deprived patients may set higher self-management standards and be more self-critical, potentially resulting in lower PAM scores.

HHD interest was not associated with PAM score in our study. Performing HD tasks requires distinct skills not measured by the PAM and HHD interest is influenced by patient, clinician and organisational-level factors. Fear of self-needling and complications and lack of suitable home support or environment influence HHD interest at the patient-level [29, 30]. Clinician

factors, such as low promotion of HHD, insufficient HHD training and perceived patient capabilities [31-33] as well as centre-level considerations such as setup costs, short-staffing and resource limitations also impact uptake [29, 34].

PatientView use did not correlate with PAM score. Previous studies show mixed results [35-39], though a systematic review found that portals are associated with greater concordance [40] and kidney patients find PatientView educational and empowering [41]. Portal use does not capture why patients accessed it or how they used the information. Patients with low activation may lack the skills to make use of the information. Simplifying portals and offering navigation support and education on applying information in daily life could enhance their potential in self-management [42-45].

Staff involvement in PAM completion may have led to higher scores due to social desirability bias [46]. Alternatively, 'co-produced' or 'mediated' PAM questionnaires result in higher scores as staff assist patients to connect their positive behaviours to the questions [46]. Consideration of completion context is therefore important when using the PAM as an outcome measure.

CSPAM and staff-level characteristics

Average CSPAM scores (mean=72.1, median=72.6) were similar to the TP-CKD study of kidney clinicians (mean=72.0, median=71.6) and the NHS England CSPAM study (mean=72.4) [47, 48]. The tendency for female staff to have higher CSPAM scores has been observed in other studies [47-50] and has been hypothesised to be due to maternalistic nature and higher representation of females in nursing and HCA roles, which are associated with higher CSPAM scores [47, 48, 51]. Doctors may have lower CSPAM scores, as found in our

study, because of higher self-critique, less time per patient and less training in person-centred care and communication than nurses and HCAs [51]. Age and experience yield mixed results in their relationship with CSPAM across studies [9, 47, 48, 52].

References

1. Gair R, Steenkamp R. Valuing Individuals: Transforming Participation in Chronic Kidney Disease Patient Activation Measure - Patient Reported Outcome Measure Report Cohort 1 [Internet]. Bristol: UK Renal Registry; 2016. [Cited 2023 Nov 12]. Available from: https://www.thinkkidneys.nhs.uk/ckd/wp-content/uploads/sites/4/2016/12/PAM-PROM-Overall-Report-Final.pdf

2. Gair R, Stannard C, Wong E, Hawkins J, Van de Veer S, Farrington K, et al. Transforming Participation in Chronic Kidney Disease - Programme report [Internet]. Bristol: UK Renal Registry; 2019. [Cited 2023 Nov 12]. Available from: https://www.thinkkidneys.nhs.uk/ckd/wp-content/uploads/sites/4/2019/01/Transforming-Participation-in-Chronic-Kidney-Disease-1.pdf

3. Ellins J, Coulte A. How engaged are people in their health care? Findings of a national telephone survey [Internet]. London: The Health Foundation; 2005. [Cited 2023 Nov 12]. Available from: https://www.health.org.uk/sites/default/files/HowEngagedArePeopleInTheirHealthcare_fullversion.pdf

4. Rademakers J, Nijman J, van der Hoek L, Heijmans M, Rijken M. Measuring patient activation in The Netherlands: translation and validation of the American short form Patient Activation Measure (PAM13). BMC Public Health [Internet]. 2012;12:577. doi: 10.1186/1471-2458-12-577.

5. Hendriks M, Rademakers J. Relationships between patient activation, disease-specific knowledge and health outcomes among people with diabetes; a survey study. BMC Health Services Research [Internet]. 2014;14(1):393. doi: 10.1186/1472-6963-14-393.

6. Graffigna G, Barello S, Bonanomi A, Lozza E, Hibbard J. Measuring patient activation in Italy: Translation, adaptation and validation of the Italian version of the patient activation measure 13 (PAM13-I). BMC Med Inform Decis Mak [Internet]. 2015;15:109. doi: 10.1186/s12911-015-0232-9

7. Bos-Touwen I, Schuurmans M, Monninkhof EM, Korpershoek Y, Spruit-Bentvelzen L, Ertugrul-van der Graaf I, et al. Patient and disease characteristics associated with activation for self-management in patients with diabetes, chronic obstructive pulmonary disease, chronic heart failure and chronic renal disease: a cross-sectional survey study. PLoS One [Internet]. 2015;10(5):e0126400. doi: 10.1371/journal.pone.0126400

8. Wilkinson TJ, Memory K, Lightfoot CJ, Palmer J, Smith AC. Determinants of patient activation and its association with cardiovascular disease risk in chronic kidney disease: A cross-sectional study. Health Expect [Internet]. 2021;24(3):843-52. doi: 10.1111/hex.13225

9. Zimbudzi E, Lo C, Ranasinha S, Fulcher GR, Jan S, Kerr PG, et al. Factors associated with patient activation in an Australian population with comorbid diabetes and chronic kidney disease: a cross-sectional study. BMJ Open [Internet]. 2017;7(10):e017695. doi: 10.1136/bmjopen-2017-017695

10. Van Bulck L, Claes K, Dierickx K, Hellemans A, Jamar S, Smets S, et al. Patient and treatment characteristics associated with patient activation in patients undergoing hemodialysis: a cross-sectional study. BMC Nephrol [Internet]. 2018;19(1):126. doi: 10.1186/s12882-018-0917-2

11. Hussein WF, Bennett PN, Sun SJ, Reiterman M, Watson E, Farwell IM, et al. Patient Activation Among Prevalent Hemodialysis Patients: An Observational Cross-Sectional Study. Journal of Patient Experience [Internet]. 2022;9:23743735221112220. doi: 10.1177/23743735221112220

12. Feil DG, Zhu CW, Sultzer DL. The relationship between cognitive impairment and diabetes self-management in a population-based community sample of older adults with Type 2 diabetes. J Behav Med [Internet]. 2012;35(2):190-9. doi: 10.1007/s10865-011-9344-6

13. Jung HP, Baerveldt C, Olesen F, Grol R, Wensing M. Patient characteristics as predictors of primary health care preferences: a systematic literature analysis. Health Expect [Internet]. 2003;6(2):160-81. doi: 10.1046/j.1369-6513.2003.00221.x

14. Cukor D, Zelnick LR, Charytan DM, Shallcross AJ, Mehrotra R. Patient Activation Measure in Dialysis-Dependent Patients in the United States. J Am Soc Nephrol [Internet]. 2021;32(12):3017-9. doi: 10.1681/ASN.2021030315

15. Magadi W, Lightfoot CJ, Memory KE, Santhakumaran S, van der Veer SN, Thomas N, et al. Patient activation and its association with symptom burden and quality of life across the spectrum of chronic kidney disease stages in England. BMC Nephrol [Internet]. 2022;23(1):45. doi: 10.1186/s12882-022-02679-w

16. Manns B, Hemmelgarn B, Lillie E, Dip SC, Cyr A, Gladish M, et al. Setting research priorities for patients on or nearing dialysis. Clin J Am Soc Nephrol [Internet]. 2014;9(10):1813-21. doi: 10.2215/CJN.01610214

17. Hussein WF, Bennett PN, Carrasco A, Sun S, Reiterman M, Watson E, et al. Changes in patient activation in people starting dialysis: A prospective longitudinal, observational study. Hemodial Int [Internet]. 2022;26(3):435-48. doi: 10.1111/hdi.13013

18. Greene J, Hibbard J, Tusler M. How much do health literacy and patient activation contribute to older adults' ability to manage their health? [Internet]. Washington DC: AARP;2005. [Cited 2023 Nov 12]. Available from: https://collections.nlm.nih.gov/catalog/nlm:nlmuid-101259851-pdf

19. Lubetkin EI, Lu WH, Gold MR. Levels and correlates of patient activation in health center settings: building strategies for improving health outcomes. J Health Care Poor Underserved [Internet]. 2010;21(3):796-808. doi: 10.1353/hpu.0.0350

20. Gwynn KB, Winter MR, Cabral HJ, Wolf MS, Hanchate AD, Henault L, et al. Racial disparities in patient activation: Evaluating the mediating role of health literacy with path analyses. Patient Educ Couns [Internet]. 2016;99(6):1033-7. doi: 10.1016/j.pec.2015.12.020

21. Baker DW, Wolf MS, Feinglass J, Thompson JA, Gazmararian JA, Huang J. Health literacy and mortality among elderly persons. Arch Intern Med [Internet]. 2007;167(14):1503-9. doi: 10.1001/archinte.167.14.1503

22. Taylor DM, Fraser S, Dudley C, Oniscu GC, Tomson C, Ravanan R, et al. Health literacy and patient outcomes in chronic kidney disease: a systematic review. Nephrol Dial Transplant [Internet]. 2018;33(9):1545-58. doi: 10.1093/ndt/gfx293

23. Bailey PK, Tomson CR, Ben-Shlomo Y. What factors explain the association between socioeconomic deprivation and reduced likelihood of live-donor kidney transplantation? A questionnaire-based pilot case-control study. BMJ Open [Internet]. 2016;6(6):e012132. doi: 10.1136/bmjopen-2016-012132

24. Wang MJ, Hung LC, Lo YT. Glycemic control in type 2 diabetes: role of health literacy and shared decision-making. Patient Prefer Adherence [Internet]. 2019;13:871-9. doi: 10.2147/PPA.S202110

25. Green JA, Mor MK, Shields AM, Sevick MA, Arnold RM, Palevsky PM, et al. Associations of health literacy with dialysis adherence and health resource utilization in patients receiving

maintenance hemodialysis. Am J Kidney Dis [Internet]. 2013;62(1):73-80. doi: 10.1053/j.ajkd.2012.12.014

26. Sheikh S, Hendry P, Kalynych C, Owensby B, Johnson J, Kraemer DF, et al. Assessing patient activation and health literacy in the ED. Am J Emerg Med [Internet]. 2016;34(1):93-6. doi: 10.1016/j.ajem.2015.09.045

27. Couture É M, Chouinard MC, Fortin M, Hudon C. The relationship between health literacy and patient activation among frequent users of healthcare services: a cross-sectional study. BMC Fam Pract [Internet]. 2018;19(1):38. doi: 10.1186/s12875-018-0724-7

28. Smith SG, Curtis LM, Wardle J, von Wagner C, Wolf MS. Skill set or mind set? Associations between health literacy, patient activation and health. PLoS One [Internet]. 2013;8(9):e74373. doi: 10.1371/journal.pone.0074373

29. Ledebo I. What limits the expansion of self-care dialysis at home? Hemodial Int [Internet]. 2008;12 Suppl 1:S55-60. doi: 10.1111/j.1542-4758.2008.00298.x

30. Walker RC, Hanson CS, Palmer SC, Howard K, Morton RL, Marshall MR, et al. Patient and caregiver perspectives on home hemodialysis: a systematic review. Am J Kidney Dis [Internet]. 2015;65(3):451-63. doi: 10.1053/j.ajkd.2014.10.020

31. McLaughlin K, Manns B, Mortis G, Hons R, Taub K. Why patients with ESRD do not select self-care dialysis as a treatment option. Am J Kidney Dis [Internet]. 2003;41(2):380-5. doi: 10.1053/ajkd.2003.50047

32. Berns JS. A survey-based evaluation of self-perceived competency after nephrology fellowship training. Clin J Am Soc Nephrol [Internet]. 2010;5(3):490-6. doi: 10.2215/CJN.08461109

33. Tennankore KK, Chan CT, Curran SP. Intensive home haemodialysis: benefits and barriers. Nat Rev Nephrol [Internet]. 2012;8(9):515-22. doi: 10.1038/nrneph.2012.145

34. Tong A, Palmer S, Manns B, Craig JC, Ruospo M, Gargano L, et al. Clinician beliefs and attitudes about home haemodialysis: a multinational interview study. BMJ Open [Internet]. 2012;2(6). doi: 10.1136/bmjopen-2012-002146

35. Roblin DW, Houston TK, 2nd, Allison JJ, Joski PJ, Becker ER. Disparities in use of a personal health record in a managed care organization. J Am Med Inform Assoc [Internet]. 2009;16(5):683-9. doi: 10.1197/jamia.M3169

36. Hibbard JH, Greene J. Who Are We Reaching Through the Patient Portal: Engaging the Already Engaged? International Journal of Person Centered Medicine [Internet]. 2011;1(4):788–93.

37. Solomon M, Wagner SL, Goes J. Effects of a Web-based intervention for adults with chronic conditions on patient activation: online randomized controlled trial. J Med Internet Res [Internet]. 2012;14(1):e32. doi: 10.2196/jmir.1924

38. Ancker JS, Osorio SN, Cheriff A, Cole CL, Silver M, Kaushal R. Patient activation and use of an electronic patient portal. Inform Health Soc Care [Internet]. 2015;40(3):254-66. doi: 10.3109/17538157.2014.908200

39. Riippa I, Linna M, Rönkkö I. A Patient Portal With Electronic Messaging: Controlled Before-and-After Study. J Med Internet Res [Internet]. 2015;17(11):e250. doi: 10.2196/jmir.4487

40. Ammenwerth E, Schnell-Inderst P, Hoerbst A. The impact of electronic patient portals on patient care: a systematic review of controlled trials. J Med Internet Res [Internet]. 2012;14(6):e162. doi: 10.2196/jmir.2238

41. Bartlett C, Simpson K, Turner AN. Patient access to complex chronic disease records on the Internet. BMC Med Inform Decis Mak [Internet]. 2012;12:87. doi: 10.1186/1472-6947-12-87

42. Sarkar U, Karter AJ, Liu JY, Adler NE, Nguyen R, Lopez A, et al. The literacy divide: health literacy and the use of an internet-based patient portal in an integrated health system-results from the diabetes study of northern California (DISTANCE). J Health Commun [Internet]. 2010;15 Suppl 2(Suppl 2):183-96. doi: 10.1080/10810730.2010.499988

43. Phelps RG, Taylor J, Simpson K, Samuel J, Turner AN. Patients' continuing use of an online health record: a quantitative evaluation of 14,000 patient years of access data. J Med Internet Res [Internet]. 2014;16(10):e241. doi: 10.2196/jmir.3371

44. Kruse CS, Argueta DA, Lopez L, Nair A. Patient and provider attitudes toward the use of patient portals for the management of chronic disease: a systematic review. J Med Internet Res [Internet]. 2015;17(2):e40. doi: 10.2196/jmir.3703

45. Irizarry T, Shoemake J, Nilsen ML, Czaja S, Beach S, DeVito Dabbs A. Patient Portals as a Tool for Health Care Engagement: A Mixed-Method Study of Older Adults With Varying Levels of Health Literacy and Prior Patient Portal Use. J Med Internet Res [Internet]. 2017;19(3):e99. doi: 10.2196/jmir.7099

46. Chew S, Brewster L, Tarrant C, Martin G, Armstrong N. Fidelity or flexibility: An ethnographic study of the implementation and use of the Patient Activation Measure. Patient Educ Couns [Internet]. 2018;101(5):932-7. doi: 10.1016/j.pec.2017.12.012

47. NHS England. How much do clinicians support patient activation? A survey of clinician attitudes and behaviours towards people taking an active role in their health and care [Internet]. 2015. [Cited 2023 Nov 12]. Available from: https://www.england.nhs.uk/wp-content/uploads/2015/11/cspam-report.pdf

48. Gair R, Steenkamp R, Caskey F, Cullen R. Valuing Individuals: Transforming Participation in Chronic Kidney Disease Clinician Support for Patient Activation Measure (CS-PAM) Survey Cohort 1 [Internet]. Bristol: UK Renal Registry; 2017. [Cited 2023 Jan 14]. Available from: https://www.thinkkidneys.nhs.uk/ckd/wp-content/uploads/sites/4/2016/10/CS-PAM-Report-FINAL.pdf

49. Stoilkova-Hartmann A, Janssen DJ, Franssen FM, Spruit MA, Wouters EF. Attitudes of healthcare professionals providing pulmonary rehabilitation toward partnership in care. Heart Lung [Internet]. 2015;44(4):347-52. doi: 10.1016/j.hrtlng.2015.05.003

50. Alvarez C, Greene J, Hibbard J, Overton V. The role of primary care providers in patient activation and engagement in self-management: a cross-sectional analysis. BMC Health Services Research [Internet]. 2016;16(1):85. doi: 10.1186/s12913-016-1328-3

51. Rademakers J, Jansen D, van der Hoek L, Heijmans M. Clinicians' beliefs and attitudes toward patient self-management in the Netherlands; translation and testing of the American Clinician Support for Patient Activation Measure (CS-PAM). BMC Health Serv Res [Internet]. 2015;15:138. doi: 10.1186/s12913-015-0799-y

52. Hibbard JH, Collins PA, Mahoney E, Baker LH. The development and testing of a measure assessing clinician beliefs about patient self-management. Health Expect [Internet]. 2010;13(1):65-72. doi: 10.1111/j.1369-7625.2009.00571.x
