# Supplementary material for: Supporting patient self-management: A cross-sectional and prospective cohort study investigating Patient Activation Measure (PAM) and Clinician Support for PAM scores as part of a multi-centre haemodialysis breakthrough series collaborative
Source: PLoS One. 2024 May 22;19(5):e0303299. doi: 10.1371/journal.pone.0303299 (PMC11111028; doi:10.1371/journal.pone.0303299)
Supplement: S1 Table — (PDF) [file pone.0303299.s005.pdf]

S1 Table. Shared HD care tasks*.

Patient Preparation	Machine Preparation and Dialysis Initiation	During and After Dialysis
Measuring weight	Lining dialysis machine	Responding to machine alarms
Measuring blood pressure and pulse	Priming dialysis machine	Disconnecting lines and completing dialysis
Measuring temperature	Programming dialysis machine	Applying pressure to needle sites or locking tunnelled line
Washing hands	Needling fistula/graft or preparing tunnelled line	Giving own anaemia injections (such as epoetin)
Preparing dressing (vascular access) pack	Connective lines to fistula/graft/tunnelled line and commencing dialysis	

*Reproduced from Fotheringham *et al.* [27]
