# Supplementary material for: Supporting patient self-management: A cross-sectional and prospective cohort study investigating Patient Activation Measure (PAM) and Clinician Support for PAM scores as part of a multi-centre haemodialysis breakthrough series collaborative
Source: PLoS One. 2024 May 22;19(5):e0303299. doi: 10.1371/journal.pone.0303299 (PMC11111028; doi:10.1371/journal.pone.0303299)
Supplement: S2 Table — (PDF) [file pone.0303299.s006.pdf]

S2 Table. Three learning events forming the basis of the BTSC.

Component	Learning Event 1 - June 2018	Learning Event 2 - October 2018	Learning Event 3 - December 2018
**Introduction**	Aims and objectives of SHAREHD.<br>Importance of clinician role and patient involvement.<br>Patient perspectives of shared care.	Reminder of aims and objectives of SHAREHD.<br>Summary of the previous learning event.	Reminder of aims and objectives of SHAREHD.<br>Summary of learning events 1 and 2.
**Data**	Explanation of PAM and the measures used in the patient questionnaires.<br>Presentation of initial patient data.	Explanation of PAM and CSPAM.<br>Presentation of initial CSPAM findings.<br>Discussion of key performance indicators.	Summary of data collection time frames.
**Top tips from kidney centres involved in the previous SHAREHD BTSC- Fotheringham *et al.* [27]**	Giving pre-dialysis patients the choice to do shared care.<br>Tailoring teaching to patients' pace and style of learning.<br>Importance of staff training, teamwork, leadership and involving HHD teams.<br>Peer-support and buddying schemes<br>Problems faced and solutions.	Kidney centres from the previous SHAREHD BTSC gave advice on communication, advertising shared care, development of patient advocate roles and presenting work at national meetings.<br>Barriers faced and achievements.	Kidney centres presented what they were most proud of, one lesson learnt, one patient engagement tip and one sustainability tip.<br>Kidney centres from the previous SHAREHD BTSC gave advice on sustainability.
**Quality Improvement**	Teams identified their stage of implementation and potential barriers and hopes.<br>Teams created their own Plan Do Study Act (PDSA) cycles, planned how they will collect data and measure progress and shared ideas.	Each kidney team presented their achievements, how they were measuring change and what PDSA cycles they had completed.	Including shared care in routine practice, job descriptions, competencies and policies.<br>Monitoring shared care.<br>Teams created a 'shared care pledge.'
**Co-production, communication and sustainability**	Teams brainstormed how they currently involve patients, potential barriers and how this could be improved.	Discussion of successful communication with stakeholders.<br>Teams made communication action plans.<br>Culture change and overcoming resistance.	The NHS Sustainability Model.<br>Overcoming resistance.<br>Teams made sustainability action plans.
