# Supplementary material for: Supporting patient self-management: A cross-sectional and prospective cohort study investigating Patient Activation Measure (PAM) and Clinician Support for PAM scores as part of a multi-centre haemodialysis breakthrough series collaborative
Source: PLoS One. 2024 May 22;19(5):e0303299. doi: 10.1371/journal.pone.0303299 (PMC11111028; doi:10.1371/journal.pone.0303299)
Supplement: S3 Table — (PDF) [file pone.0303299.s007.pdf]

S3 Table. Patient characteristics, univariate analyses and final multivariable model predicting PAM score.

Baseline patient-level characteristics (n=283)†		Univariate analyses (n=236)		Final multivariable analysis (n=197) R^2=0.284	
**Variables**	**Number (%)**	**Mean PAM score (SD)‡**	**P value**	**Regression Coefficient (95% CI)**	**P value**
**Age**			0.028*		
<50	58 (20.5)	59.3 (13.2)		4.850 (0.884 to 8.816)	0.017*
≥50	225 (79.5)	54.6 (11.2)		(Ref)	
Missing	0 (0)				
**Who completed YHS2**			0.035*		
On their own	151 (53.4)	54.7 (10.6)		(Ref)	
With staff	101 (35.7)	57.7 (13.2)		5.493 (1.884 to 9.102)	0.003*
With friend/relative	26 (9.2)	51.3 (9.7)		1.525 (-4.827 to 7.876)	0.636
Missing	5 (1.8)				
**Health literacy**			<0.001*		
Limited	129 (45.6)	52.4 (10.8)		-6.151 (-9.544 to -2.758)	<0.001*
Adequate	147 (51.9)	58.3 (11.7)		(Ref)	
Missing	7 (2.5)				
**Adjusted UK Index of Multiple Deprivation Quintiles**			0.064		
1 – Least deprived	22 (7.8)	55.5 (10.4)		-2.884 (-8.641 to 2.873)	0.324
2	30 (10.6)	56.5 (11.8)		-1.971 (-7.031 to 3.088)	0.443
3	33 (11.7)	59.4 (14.2)		4.155 (-0.489 to 8.799)	0.079
4	47 (16.6)	51.1 (10.6)		-5.900 (-10.148 to -1.653)	0.007**
5 – Most deprived	116 (41.0)	56.4 (11.7)		(Ref)	
Missing	35 (12.4)				
**Where YHS2 completed**			0.223		
Clinical environment	223 (78.8)	55.9 (12.3)			
At home	55 (19.4)	53.9 (8.7)			
Missing	5 (1.8)				
**Use of PatientView**			0.874		
Yes	57 (20.1)	55.0 (11.5)			
No	187 (66.1)	55.5 (11.8)			
Don't know	32 (11.3)	56.4 (11.5)			
Missing	7 (2.5)				
**HHD interest**			0.311		
Yes	28 (9.9)	57.7 (14.2)			
No	207 (73.1)	55.7 (11.9)			
Unsure	36 (12.7)	53.3 (9.3)			
Missing	12 (4.2)				
**EQ-5D-5L Problems with mobility**			0.001*		
None/slight	143 (50.5)	57.5 (11.0)		(Ref)	
Mod/Sev/Ov	135 (47.7)	52.7 (11.6)		-1.689 (-5.499 to 2.122)	0.383
Missing	5 (1.8)				

**EQ-5D-5L Problems with self-care**			<0.001*		
None/slight	220 (77.7)	56.7 (11.6)		(Ref)	
Mod/Sev/Ov	58 (20.4)	49.9 (9.3)		-5.591 (-10.383 to -0.798)	0.022*
Missing	5 (1.8)				
**EQ-5D-5L Problems with usual activities**			0.073		
None/slight	147 (51.9)	56.6 (11.9)		(Ref)	
Mod/Sev/Ov	132 (46.6)	53.9 (11.1)		3.227 (-0.509 to 6.963)	0.090
Missing	4 (1.4)				
**EQ-5D-5L Pain/discomfort**			0.037*		
None/slight	173 (61.1)	56.4 (11.3)		(Ref)	
Mod/Sev/Ov	102 (36.1)	53.1 (11.4)		-0.088 (-3.930 to 3.753)	0.964
Missing	8 (2.8)				
**EQ-5D-5L Anxiety/ depression**			0.018*		
None/slight	205 (72.4)	56.3 (11.4)			
Mod/Sev/Ov	71 (25.2)	52.2 (11.0)			
Missing	7 (2.5)				
**Itching**			0.007*		
Absent	127 (44.9)	57.6 (12.0)		(Ref)	
Mild/Mod/Sev/Ov	151 (53.4)	53.5 (10.9)		-2.308 (-5.504 to 0.888)	0.156
Missing	5 (1.8)				
**Difficulty sleeping**			0.091		
Absent	111 (39.2)	57.0 (11.4)		(Ref)	
Mild/Mod/Sev/Ov	165 (58.3)	54.3 (11.6)		-0.408 (-3.771 to 2.956)	0.811
Missing	7 (2.5)				
**Changes in skin**			0.018*		
Absent	157 (55.5)	56.9 (12.2)		(Ref)	
Mild/Mod/Sev/Ov	120 (42.4)	53.4 (10.4)		-0.600 (-4.041 to 2.842)	0.731
Missing	6 (2.1)				
**Feeling anxious/worried about illness/treatment**			0.025*		
Absent	136 (48.1)	57.2 (11.7)		(Ref)	
Mild/Mod/Sev/Ov	142 (50.2)	53.8 (11.2)		-1.183 (-4.753 to 2.387)	0.514
Missing	5 (1.8)				
**Feeling depressed**			0.041*		
Absent	148 (52.3)	56.9 (11.7)		(Ref)	
Mild/Mod/Sev/Ov	130 (45.9)	53.8 (11.2)		-0.822 (-4.638 to 2.994)	0.671
Missing	5 (1.8)				
**PAM levels (n=236)†**					
1	55 (23.3)				
2	94 (39.8)				
3	57 (24.2)				
4	30 (12.7)				

**All staff centre-level mean CSPAM score at baseline**			0.328 (-0.157 to 0.812)	0.184

*P<0.05
Symptoms P>0.1 on univariate analysis are detailed separately in S3 Table, due to the large number of patient symptoms in POS-S Renal.
†n=283, apart from PAM levels, where n=236 after excluding patients responding with all ‘agree strongly’ (n=14), all ‘disagree strongly’ (n=2) and who had at least one missing response (n=31).
‡SD = standard deviation
