# Supplementary material for: Supporting patient self-management: A cross-sectional and prospective cohort study investigating Patient Activation Measure (PAM) and Clinician Support for PAM scores as part of a multi-centre haemodialysis breakthrough series collaborative
Source: PLoS One. 2024 May 22;19(5):e0303299. doi: 10.1371/journal.pone.0303299 (PMC11111028; doi:10.1371/journal.pone.0303299)
Supplement: S4 Table — (PDF) [file pone.0303299.s008.pdf]

S4 Table. Symptoms with P>0.1 on univariate analysis predicting PAM score.

Baseline patient-level characteristics (n=283)		Univariate analysis (n=236)	
**Variables**	**Number (%)**	**Mean PAM score (SD)**	**P value**
**Pain**			0.370
Absent	125 (44.2)	56.1 (10.8)	
Mild/Mod/Sev/Ov	146 (51.6)	54.7 (12.1)	
Missing	12 (4.2)		
**Shortness of breath**			0.338
Absent	116 (41.0)	56.0 (12.4)	
Mild/Mod/Sev/Ov	153 (54.1)	54.6 (10.9)	
Missing	14 (4.9)		
**Nausea**			0.607
Absent	167 (59.0)	55.7 (11.5)	
Mild/Mod/Sev/Ov	111 (39.2)	54.9 (11.7)	
Missing	5 (1.8)		
**Vomiting**			0.375
Absent	214 (75.6)	55.7 (11.4)	
Mild/Mod/Sev/Ov	63 (22.3)	54.1 (12.3)	
Missing	6 (2.1)		
**Poor appetite**			0.654
Absent	145 (51.2)	55.7 (11.6)	
Mild/Mod/Sev/Ov	132 (46.6)	55.0 (11.6)	
Missing	6 (2.1)		
**Constipation**			0.756
Absent	174 (61.1)	55.2 (11.5)	
Mild/Mod/Sev/Ov	100 (35.3)	55.7 (11.8)	
Missing	9 (3.2)		
**Sore or dry mouth**			0.164
Absent	148 (52.3)	56.2 (12.5)	
Mild/Mod/Sev/Ov	127 (44.9)	54.1 (10.3)	
Missing	8 (2.8)		
**Drowsiness**			0.469
Absent	119 (42.0)	56.0 (11.6)	
Mild/Mod/Sev/Ov	159 (56.2)	54.9 (11.5)	
Missing	5 (1.8)		
**Poor mobility**			0.254
Absent	77 (27.2)	56.8 (11.2)	
Mild/Mod/Sev/Ov	194 (68.6)	54.8 (11.6)	
Missing	12 (4.2)		

Baseline patient-level characteristics (n=283)		Univariate analysis (n=236)	
**Variables**	**Number (%)**	**Mean PAM score (SD)**	**P value**
**Restless legs**			0.803
Absent	137 (48.4)	55.0 (11.2)	
Mild/Mod/Sev/Ov	138 (48.8)	55.3 (11.5)	
Missing	8 (2.8)		
**Diarrhoea**			0.673
Absent	189 (66.8)	55.0 (11.5)	
Mild/Mod/Sev/Ov	83 (29.3)	55.7 (11.8)	
Missing	11 (3.9)		
**Weakness/lack of energy**			0.152
Absent	40 (14.1)	52.4 (11.1)	
Mild/Mod/Sev/Ov	237 (83.7)	55.7 (11.6)	
Missing	6 (2.1)		

Mod = Moderate
Sev = Severe
Ov = Overwhelming
