# Supplementary material for: Supporting patient self-management: A cross-sectional and prospective cohort study investigating Patient Activation Measure (PAM) and Clinician Support for PAM scores as part of a multi-centre haemodialysis breakthrough series collaborative
Source: PLoS One. 2024 May 22;19(5):e0303299. doi: 10.1371/journal.pone.0303299 (PMC11111028; doi:10.1371/journal.pone.0303299)
Supplement: S5 Table — (PDF) [file pone.0303299.s009.pdf]

S5 Table. Comparison of staff-level characteristics in paired and unpaired univariate analyses.

Staff-level characteristics	Paired analyses		Unpaired analyses	
	Main analysis (n=37)	Sensitivity analysis (n=26)	Main analysis (n=174)	Sensitivity analysis (n=199)
**Age (years)**				
<35	4 (10.8)	4 (15.4)	36 (20.7)	41 (20.6)
35-44	14 (37.8)	8 (30.8)	63 (36.2)	68 (34.2)
45-54	13 (35.1)	9 (34.6)	58 (33.3)	70 (35.2)
>=55	6 (16.2)	5 (19.2)	17 (9.8)	20 (10.1)
**Gender**				
Male	8 (21.6)	5 (19.2)	30 (17.2)	33 (16.6)
Female	29 (78.4)	21 (80.8)	143 (82.2)	165 (82.9)
Other	0 (0)	0 (0)	1 (0.6)	1 (0.5)
**Staff type**				
Doctor	10 (27.0)	7 (26.9)	32 (18.4)	34 (17.1)
Nurse	21 (56.8)	15 (57.7)	103 (59.2)	120 (60.3)
HCA	2 (5.4)	0 (0)	26 (14.9)	31 (15.6)
AHP and other	4 (10.8)	4 (15.4)	13 (7.5)	14 (7.0)
**Years working with patients with chronic disease**				
0-5	4 (10.8)	4 (15.4)	34 (19.5)	36 (18.1)
6-10	2 (5.4)	2 (7.7)	24 (13.8)	30 (15.1)
11-15	13 (35.1)	8 (30.8)	43 (24.7)	52 (26.1)
16-20	9 (24.3)	5 (19.2)	31 (17.8)	32 (16.1)
>20	9 (24.3)	7 (26.9)	42 (24.1)	49 (24.6)

Figures given as frequency (percentage)
