# Supplementary material for: Supporting patient self-management: A cross-sectional and prospective cohort study investigating Patient Activation Measure (PAM) and Clinician Support for PAM scores as part of a multi-centre haemodialysis breakthrough series collaborative
Source: PLoS One. 2024 May 22;19(5):e0303299. doi: 10.1371/journal.pone.0303299 (PMC11111028; doi:10.1371/journal.pone.0303299)

S1 Fig. Stacked bar charts of categorised baseline centre-level percentage of staff with a high CSPAM level for all staff and nurses and HCAs only by PAM level. Figures within stacks represent the percentage of patients within each level. Patient N = number of patients represented in each column. (A) Chi-squared for all PAM levels: P=0.478, Chi-squared for PAM level 4: P=0.056. (B) Chi-squared for all PAM levels: P=0.901, Chi-squared for PAM level 4: P=0.101.

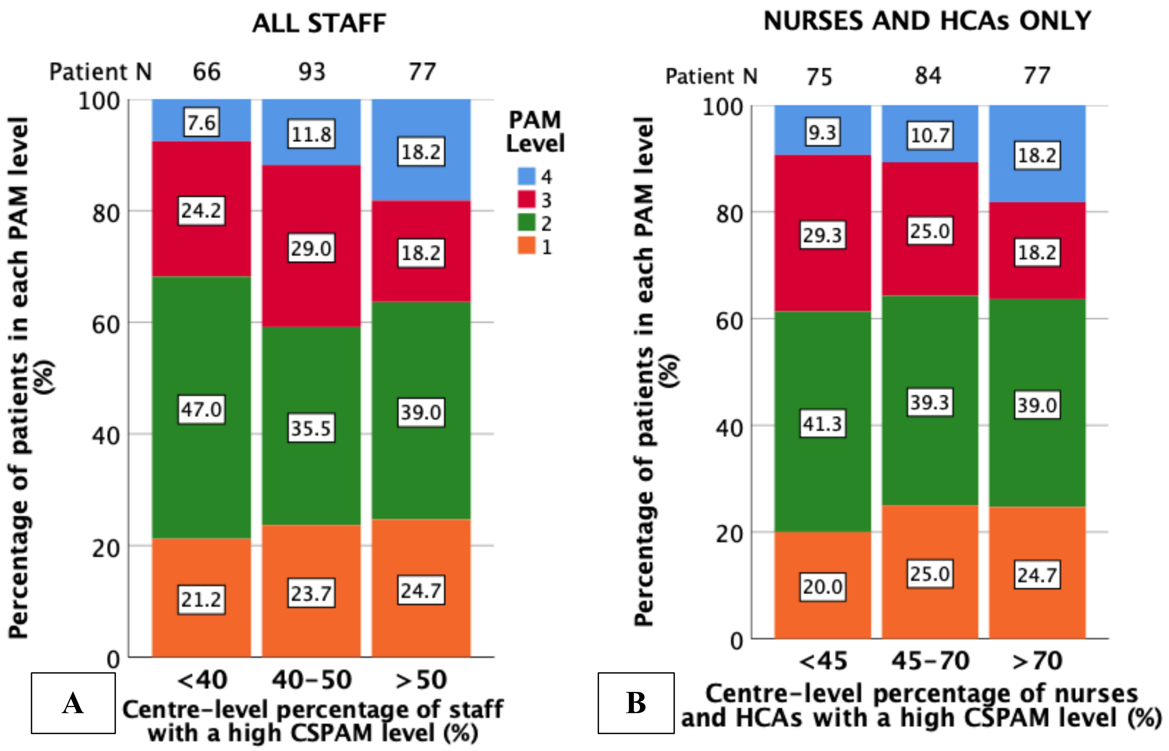

Supplement: S1 Fig — Figures within stacks represent the percentage of patients within each level. Patient N = number of patients represented in each column. (A) Chi-squared for all PAM levels: P = 0.478, Chi-squared for PAM level 4: P = 0.056. (B) Chi-squared for all PAM levels: P = 0.901, Chi-squared for PAM level 4: P = 0.101. (PDF) [file pone.0303299.s010.pdf]
